# Exploring Synergistic Inhibition of Inflammatory and Antioxidant Potential: Integrated *In Silico* and *In Vitro* Analyses of *Garcinia mangostana*, *Curcuma comosa*, and *Acanthus ebracteatus*

**DOI:** 10.1155/2024/8584015

**Published:** 2024-09-18

**Authors:** Komgrit Eawsakul, Kingkan Bunluepuech

**Affiliations:** ^1^ College of Graduate Studies Walailak University, Nakhon Si Thammarat 80160, Thailand; ^2^ Applied Thai Traditional Medicine School of Medicine Walailak University, Nakhon Si Thammarat 80160, Thailand; ^3^ Research Excellence Center for Innovation and Health Products (RECIHP) Walailak University, Nakhon Si Thammarat 80160, Thailand

## Abstract

Compared with the use of a single herb alone, herb combinations can significantly increase their effectiveness in treating various diseases. The objective of this research was to determine the appropriate proportions of *Garcinia mangostana* (*GM*), *Curcuma comosa* (*CC*), and *Acanthus ebracteatus* (*AE*) to enhance their antioxidant and anti-inflammatory properties. The study employed computer modeling (*in silico*) and laboratory testing (*in vitro*). The optimal levels of phenolic and flavonoid compounds were achieved with a ratio of 15 parts *GM*, 5 parts *CC*, and 10 parts *AE*. In contrast, the 10 : 15 : 5 ratio resulted in the highest antioxidant activity, reducing DPPH radicals by 80.33% and ABTS radicals by 92.20%. The ratio had a synergistic effect and was within the safe range, ranging from 82.97 to 147.04 *µ*g/mL. The ratio of 5 : 10 : 15 had the greatest anti-inflammatory effects, with an exceptional activity level of 98.86%. It effectively suppressed 23 genes or proteins, including the prominent NFE2L2 gene, and had a synergistic effect. This combination effectively inhibits inflammation (IC_50_: 12.05 *µ*g/mL) and is safe for macrophages at concentrations up to 50 *µ*g/mL. These findings emphasize the potential of these herbal mixtures for antioxidant and anti-inflammatory applications.

## 1. Introduction

The use of herbs is prevalent in many developing countries, and historically, herbs have been commonly used for treating diseases in developed countries as well [1]. This widespread acceptance underscores the long-standing tradition of herbal treatments in healthcare [2]. In regions where modern medicine may be limited, herbs are frequently used to alleviate pain and inflammation [3]. A World Health Organization (WHO) [4] survey revealed that more than 75% of people worldwide depend on herbal remedies for their medical needs, highlighting the enduring significance of traditional therapies in global health. Botanical sources of natural resources account for up to 85% of the medications used globally for basic healthcare [5]. Recent research has emphasized the efficacy of natural substances in diverse medical applications, indicating their promise in addressing multiple health conditions [6]. There is a global abundance of approximately 70,000 distinct species of medicinal plants, offering a diverse array of alternative remedies for a multitude of ailments [7]. The development of pharmacological drugs that target numerous biological objectives requires the use of both natural and synthetic resources. This exemplifies the diverse and intricate methods employed in drug discovery [8]. Empirical evidence strongly suggests that drugs sourced from natural compounds are typically more effective and safer [9]. Recent scientific research has increasingly focused on exploring the analgesic and anti-inflammatory properties of medicinal plants [10], underscoring a growing interest in botanical remedies. In today's evolving landscape, the importance of natural substances in pharmacology is emphasized, offering promising avenues for developing targeted therapies to alleviate pain and inflammation [11].

Lipopolysaccharide (LPS) from Gram-negative bacteria triggers macrophage inflammation, leading to the release of key inflammatory mediators such as TNF-*α*, IL-6, nitric oxide (NO), and prostaglandin E2 (PGE2). Controlling macrophage activation is crucial for treating various inflammatory conditions. While pharmaceuticals such as corticosteroids, immunosuppressants, biologics, and NSAIDs are standard treatments, prolonged NSAID use can cause significant side effects, including gastrointestinal, renal, and cardiovascular complications. Given these limitations, interest in exploring medicinal plants, which are rich in anti-inflammatory and analgesic phytochemicals, is increasing. These natural compounds offer potential alternatives to traditional therapies, with ongoing research aimed at isolating and identifying bioactive constituents that can effectively combat inflammation and pain.

The tropical plant *Garcinia mangostana* Linn. sometimes referred to as mangosteen, is indigenous to regions such as Sri Lanka, Myanmar, India, Thailand, and Malaysia. The plants gradually grow, reaching heights of 6 to 25 meters. It has durable leaves with a smooth texture [12]. The fruit is famous for its highly aromatic scent and its deep purple or reddish skin, which encloses a white, succulent pulp that achieves a harmonious balance between sweetness and acidity [13]. Mangosteen is commonly referred to as the “queen of fruits” because of its extraordinary taste, which is highly valued in Southeast Asia. The pericarp of this plant has historically been utilized in indigenous medicine to treat amoebic dysentery and wounds [14]. In addition, it has been employed in Ayurvedic medicine to address illnesses such as cholera and inflammatory disorders [15]. *Curcuma comosa*, commonly referred to as “Waan Chak Mod Look” in Thai, has historical importance as both a culinary component and a treatment for postpartum uterine bleeding and digestive problems. The rhizomes of this plant exhibit a wide range of biological activities, such as antioxidant, anti-inflammatory, and estrogenic effects [16–18]. *Acanthus ebracteatus* Vahl, commonly known as Sea Holly, is distributed in mangrove environments in Vietnam, the Philippines, Indonesia, Malaysia, and Thailand [19]. This plant belongs to the Acanthaceae family and is well known for its medicinal properties in treating dermatological difficulties, menstrual irregularities, wounds, and a range of other maladies, such as parasitic infections, hepatitis, asthma, and rheumatic conditions [20].

Compared with single-herb treatments, polyherbal formulations, which combine multiple medicinal plants, have gained significant attention in recent years because of their enhanced therapeutic effects. This synergistic approach is particularly beneficial in managing complex conditions such as inflammation, where multiple pathways are involved. The rationale behind using polyherbal formulations lies in the complementary actions of different phytochemicals, which work together to enhance efficacy, reduce toxicity, and target multiple aspects of a disease simultaneously [21]. In this study, we focused on the combined use of three botanicals, each known for its potent anti-inflammatory properties: *Garcinia mangostana* Linn., *Curcuma comosa*, and *Acanthus ebracteatus* Vahl. While these plants have been individually studied for their therapeutic effects, exploration of their combined, synergistic effects remains limited. Previous research has shown that polyherbal formulations can significantly improve outcomes in conditions such as diabetic nephropathy, where oxidative stress and inflammation play critical roles [22]. The combination of these plants is hypothesized to provide superior anti-inflammatory effects because of the diverse array of bioactive compounds that can act on various inflammatory pathways. By targeting multiple mechanisms simultaneously, polyherbal formulations not only enhance efficacy but also reduce the potential for adverse effects that may arise from higher doses of single compounds. This study aimed to investigate the synergistic effects of these botanicals, with a focus on their combined impact on inflammation and oxidative stress, by using LC-MS/MS to analyze their phytochemical composition and employing molecular docking and network pharmacology to explore synergistic interactions among bioactive compounds.

## 2. Materials and Methods

### 2.1. Plant Material

To conduct this investigation, samples of leaves of *Acanthus ebracteatus* (*AE*), pericarp of *Garcinia mangostana* (*GM*), and rhizomes of *Curcuma comosa* (*CC*) sourced from Vejpongosot Pharmaceutical Company of Thailand were procured. Voucher samples corresponding to CC, AE, and GM were assigned TTM-c Nos. 1000807, 1000808, and 1000809, respectively, and were subsequently deposited at the Thai Traditional Medicine Herbarium, Department of Thai Traditional and Alternative Medicine, Ministry of Public Health, Bangkok, Thailand. The acquired botanical materials underwent desiccation and were subsequently subjected to sieve and pulverize, resulting in an average particulate size of 105 mm. The processed samples were then stored in opaque, hermetically sealed polyethylene bags under refrigeration at a temperature of 4°C until the extraction procedures commenced.

### 2.2. Chemicals and Reagents

Ethanol (purity: 95%, molecular weight: 46.07 g/mol), aluminum chloride salt (purity: ≥99%, molecular weight: 133.34 g/mol), methanol (purity: ≥99.9%, molecular weight: 32.04 g/mol), sodium carbonate anhydrous (purity: ≥99.5%, molecular weight: 105.99 g/mol), Folin–Ciocalteu phenol reagent, which consists of phosphomolybdic acid (H₃PMo₁₂O₄₀, molecular weight: 1825.25 g/mol) and phosphotungstic acid (H₃PW₁₂O₄₀, molecular weight: 2880.05 g/mol) in an acidic solution, quercetin (purity: ≥95%, molecular weight: 302.24 g/mol), 2,2-diphenyl-picrylhydrazyl (DPPH) (purity: ≥95%, molecular weight: 394.32 g/mol), and 2,2′-azino-bis-3-ethylbenzothiazoline-6-sulfonic acid (ABTS+•) (purity: ≥98%, molecular weight: 548.68 g/mol) were purchased from Sigma-Aldrich. All chemicals and reagents used in this study were of analytical grade.

### 2.3. Extraction Process

In the initial extraction method, predetermined quantities of desiccated plant materials, as shown in Table 1, were macerated in boiling water for 30 minutes before filtration. Upon cooling, the filtrate was subjected to freeze-dry [23]. The isolation of plant constituents was accomplished by using ethanol as the solvent. The extraction process involved the maceration technique and was extended over a period of three days. The resulting mixture was subsequently filtered using Whatman No. 1 filter paper. The resulting filtrate was then concentrated using a rotary evaporator (Heidolph Basic Hei-VAP ML, Schwabach, Germany) to yield a highly viscous ethanol-soluble extract. To ensure optimal extraction efficiency, we repeated the maceration process twice under identical conditions. The residual ethanol in the herbal material was subsequently removed through further evaporation using a vacuum drying apparatus (Binder VD 23, Tuttlingen, Germany) set at a drying temperature of 85°C and a vacuum pressure of 46 kPa. Evaporation was continued until a consistent weight was attained, indicative of complete ethanol removal [24].

### 2.4. Analysis of the Extract Yield

The percentage yield of the extract [25] was calculated using equation (1). This calculation process was replicated for powdered formulations as well as for individual botanicals extracted by boiling water and ethanol solvents.

(1) $$\begin{array}{c} \text{Percentage yield}=\frac{W_{2}\;–\;W_{1}}{W_{0}}\times 100, \end{array}$$

where “*W*_0_” represents the mass of the desiccated sample, “*W*_1_” denotes the mass of the receptacle, and “*W*_2_” signifies the combined mass of the reconstituted extract and the receptacle.

### 2.5. Phenolic Compound Contents

The total phenolic content was measured using the Folin‒Ciocalteu method, as described in a previous study [23]. To prepare the extracts, a stock solution was diluted with either distilled water or ethanol to reach a concentration of 1 mg/mL. Then, 100 *µ*L of a 0.1 M Na_2_CO_3_ solution and 100 *µ*L of a 10% Folin–Ciocalteu reagent were added to each well of a 96-well plate. The plate was left at room temperature for an hour. After incubation, the absorbance of the mixture was read at 750 nm. Gallic acid solutions, with concentrations ranging from 6.25 to 800 *µ*g/mL, were used to create a standard curve. The phenolic content was calculated as gallic acid equivalents (GAEs) in milligrams per gram of dried plant extract.

### 2.6. Flavonoid Compound Content

The total flavonoid content in the extract was quantified using a well-established protocol [23, 26]. Briefly, the extracts (1 mg/mL) or quercetin standard solutions (6.25 to 200 *µ*g/mL) were mixed with 100 *µ*L of a 2% AlCl_3_ solution in methanol and incubated at room temperature for 30 minutes. Afterward, the absorbance of the mixture was measured at 415 nm against a blank. The results are expressed as quercetin equivalents (QEs) per gram of dried plant extract, indicating the total flavonoid content.

### 2.7. Chemical Compound Analysis via LC-ESI-QTOF-MS/MS

The chemical composition of the herbal formulation was analyzed via liquid chromatography-electrospray ionization quadrupole time-of-flight mass spectrometry (LC-ESI-QTOF-MS/MS) [25]. LC separation was performed with an Agilent 1260 Infinity Series HPLC system with a Luna C-18 column (5 *µ*m particle size, 4.6 mm × 150 mm; Phenomenex Inc., Torrance, USA). The mobile phase consisted of acetonitrile with 0.1% formic acid (v/v) in both phases A (aqueous) and B (acetonitrile), employing a linear gradient from 5% to 95% A over 30 minutes. Each injection (10 *μ*L) was followed by a 10-minute re-equilibration, while maintaining a constant flow rate of 0.5 mL/min. Mass spectrometric analysis was performed on an Agilent 6540 ultrahigh-definition accurate mass Q-TOF instrument operating in positive and negative ESI modes within the m/z range of 100–1000 Da. The data were processed using Agilent Mass Hunter Qualitative Analysis and Data Acquisition Software B.05.01.

### 2.8. 2,2-Diphennyl-1-picrylhydrazyl (DPPH) Radical Scavenging Activity

Following a standardized method [23], the ability of the extract to scavenge free radicals was evaluated. Briefly, 20 *µ*L of the extract or a Trolox solution in ethanol was mixed with 180 *µ*L of a freshly prepared DPPH solution. The solution was vigorously agitated, placed in an incubator at ambient temperature, and shielded from light. The absorbance was measured at a wavelength of 517 nm after a 30-minute incubation period. The operation was iterated thrice to guarantee precision. The results were quantified as Trolox equivalents (TEs) per gram of the plant extract that had been dried. The DPPH scavenging activity was calculated using the following equation to determine the outcomes.

(2) $$\begin{array}{c} \%\text{Scavenging activity}=\frac{\text{Abs of control }-\;\left(\text{Abs of sample }-\text{ Abs of blank}\right)}{\text{Abs of control}}\times 100. \end{array}$$

A graph depicting the activity of free radical scavenging was subsequently generated to determine the IC_50_ value, denoting the dose required to inhibit 50% of the DPPH activity.

### 2.9. Activity of ABTS.+ Scavenging

The extract's capacity to scavenge free radicals was assessed using the ABTS decolorization assay [27]. The combination was kept in the dark at room temperature for 12–16 hours, resulting in the formation of the ABTS radical cation (ABTS.+) by adding 2.45 millimolar potassium persulfate to a 7 millimolar ABTS solution. The ABTS.+ reagent was further diluted with methanol until it reached a final absorbance of 0.70 ± 0.02. An incubation period of 45 minutes was carried out using a mixture of 20 *µ*L of the extract or Trolox and 180 *µ*L of the ABTS.+ reagent. The experiments were replicated a minimum of three times. The decrease in absorbance at 734 nm was calculated as a percentage using the following equation to determine the extent of inhibition.

(3) $$\begin{array}{c} \%\text{Scavenging activity}=\frac{\text{Abs of control }-\;\left(\text{Abs of sample }-\text{ Abs of blank}\right)}{\text{Abs of control}}\times 100. \end{array}$$

IC_50_, which indicates the concentration at which ABTS.+ activity is inhibited by 50%, was determined by constructing and analyzing a concentration-response curve for ABTS.+.

### 2.10. Anti-Inflammatory Activity

#### 2.10.1. Stimulation of RAW 264.7 Cells with LPS

Following a 24-hour incubation period, the DMEM was removed from the 96-well plate containing RAW 264.7 cells. To amplify the effects of LPS induction, the cells were subjected to LPS at doses of 1, 3, and 5 *μ*g/mL. A concentration of 1 *μ*g/mL LPS was the most effective at stimulating RAW 265.7 cells, resulting in a much higher rate of induction than other concentrations.

#### 2.10.2. Determination of Nitric Oxide Concentrations

This study was conducted to examine the anti-inflammatory properties of herbal mixtures and individual herbs using the murine macrophage cell line RAW 264.7. The cells were placed in a 96-well plate at a concentration of 1 × 10^5^ cells per well and left to attach for 24 hours. After the cells were incubated, LPS (1 *μ*g/mL) was introduced to stimulate the RAW 264.7 cells, causing the generation of nitrite in the medium as an indicator of the inflammatory response. The activated cells were subsequently subjected to a 24-hour treatment with either a mixture of herbal substances or individual herbs. The quantity of nitrite in the culture supernatants, which indicates the metabolism of nitric oxide (NO), was evaluated using the Griess reagent [28]. The anti-inflammatory effects were evaluated by calculating the percentage of NO inhibition using equation (4). The studies were performed three times, and the results are reported as the average value plus or minus the standard deviation.

(4) $$\begin{array}{c} \%\text{ NO inhibition}=\frac{\text{ODcontrol }–\text{ ODsample}}{\text{ODcontrol}}\times 100. \end{array}$$

### 2.11. Synergistic Effect Analysis

One commonly used method for evaluating the effectiveness of mixed plant extracts involves calculating the fractional percentage inhibitory index (FPI) [29]. This indicator is used to ascertain the interaction between medicines, namely, if they exhibit synergy, additivity, or antagonism. The inhibitory concentration was determined by measuring the percentage of the inhibition response. The FPI is calculated using equation (5). Synergism is observed when the fractional percentage inhibitory index (FPI) of the combination is equal to or greater than 2.0. Additivity is suggested when the FPI falls within the range of 0.5 to 2.0. A number below 0.5 indicates antagonism. Further verification was carried out using Comsyn software version 1.0 to guarantee precision and mitigate computational inaccuracies. The criteria for evaluating interactions are defined as follows: synergism is confirmed if the combination index (CI) is less than 1, an additive effect is confirmed if the CI equals 1, and antagonism is confirmed if the CI exceeds 1 [30].

(5) $$\begin{array}{c} \text{Fractional percentage inhibitory index}=\frac{\left(\%\text{ Inhibition of combination}\right)}{\left(\%\text{ Inhibition of }A+\%\text{ Inhibition of }B+\%\text{ Inhibition of }C\right)}. \end{array}$$

### 2.12. Selectivity Indices for Anti-Inflammatory Studies

To evaluate the safety and efficacy of the herbal formulation, the selectivity index (SI) was calculated. The SI provides a measure of the therapeutic window by comparing the cytotoxic effects on fibroblasts with the desired anti-inflammatory activity in Raw 264.7 cells. First, the IC_50_ value, which represents the concentration required to achieve 50% inhibition of the inflammatory response, was determined for the herbal formulation against Raw 264.7 cells. This IC_50_ value is critical for understanding the ability of the formulation to suppress inflammation. Then, the IC_20_ value for cytotoxicity was determined using L929 fibroblasts. This value indicates the concentration at which the herbal formulation inhibits or kills 20% of the fibroblast population, providing insight into the potential cytotoxic effects of the formulation on fibroblasts. The selectivity index (SI) was then calculated as the ratio of the IC_20_ for fibroblast cytotoxicity to the IC_50_ for anti-inflammatory activity, as shown in the following equation:

(6) $$\begin{array}{c} \text{SI}=\frac{{\text{IC}}_{20}\text{ for Cytotoxicity}\left(\text{Fibroblast}\right)}{{\text{IC}}_{50}\text{ for Anti}-\text{inflammatory}\left(\text{Raw }264.7\text{ cells}\right)}. \end{array}$$

An SI value greater than 1 suggests that the formulation is more selective in its anti-inflammatory action and is less toxic to fibroblasts, indicating a favorable safety profile.

### 2.13. Cell Culture of L929 Fibroblasts

Murine L929 fibroblasts sourced from the Japanese Collection of Research Bioresources (JCRB) Cell Bank were cultured in Dulbecco's modified Eagle's medium (DMEM, Gibco, USA) supplemented with 10% fetal bovine serum (FBS, Gibco, USA) and 1,000 U/mL penicillin plus 100 *µ*g/mL streptomycin (Gibco, USA) and maintained at 37°C in a 5% CO_2_ atmosphere. Upon reaching approximately 80% confluence, the cells were subcultured with trypsin-EDTA solution and prepared for experimental use.

### 2.14. *In Vitro* Cytotoxicity

Prior to commencing the research, approval for the use of the L929 cell line (clearing number: WU-IBC-67-010) was granted by the Institutional Biosafety Committee (IBC) of Walailak University. The cytotoxic effects of herbal recipes and individual herbs on L929 fibroblasts were evaluated using the MTT assay [31]. L929 cells were seeded onto 24-well culture plates and incubated under standard conditions at 37°C with 5% CO_2_ for 24 hours. The cells were subsequently treated with various concentrations of samples (7.81−1,000 *µ*g/mL) and the corresponding controls (untreated), followed by an additional 24-hour incubation period. After they were washed with phosphate-buffered saline (PBS), the cells were exposed to the MTT solution (0.5 mg/mL) for 3 hours, leading to the formation of insoluble formazan crystals. These crystals were then dissolved in 100 *µ*L of the dimethyl sulfoxide (DMSO) solution, and the absorbance of each sample was measured at 570 nm using a microplate reader (Tecan, Infinite 200 Pro M Plex, Switzerland).

### 2.15. Screening of the Recipe's Primary Active Components and Targeting of the Recipe

The principal constituents of the herbal formulation were identified via liquid chromatography-electrospray ionization quadrupole time-of-flight mass spectrometry (LC-ESI-QTOF-MS/MS) analysis. Compounds meeting the screening criteria, defined as having a relative abundance equal to or exceeding 1% and a match score of 90 or higher [32], were considered significant constituents. The pivotal bioactive components present in the herbal formulation were assessed for their drug-likeness properties using Lipinski's rule of five, a widely employed guideline for predicting drug bioavailability and permeability. This evaluation was conducted using the SwissADME online platform (https://www.swissadme.ch). In addition, the potential target proteins linked to the identified main active chemicals were determined via computational target prediction methods. To achieve this goal, the SwissTargetPrediction tool (https://www.swisstargetprediction.ch/) was used to predict the molecular targets that interact with the detected compounds; this provides vital information on the pharmacological mechanisms that are responsible for the observed biological effects.

### 2.16. Target Accumulation with Anti-Inflammatory and Antioxidant Properties

Potential targets with antioxidant and anti-inflammatory properties were obtained from the GeneCards database (https://www.genecards.org/) [33] and the DrugBank database (https://go.drugbank.com) [34] for clarification. The search criteria utilized the terms “oxidative stress” and “inflammation” to ascertain pertinent targets. To ensure consistency and efficiency, any redundant entries were systematically eliminated using Microsoft Excel 2019 (Redmond, WA, USA).

### 2.17. Network Analysis of the Main Active Herbal Recipe Targets

Intersections between the target proteins associated with anti-inflammatory or antioxidant properties and the main active herbal recipe were generated using Venny 2.1 (https://bioinfogp.cnb.csic.es/tools/venny/index.html), followed by the construction of a network using Cytoscape (version 3.7.1) [35]. The target proteins are depicted as pink nodes, whereas the active components of the herbal recipe are represented as green nodes [36].

### 2.18. Pathway Enrichment Analysis for Kyoto Encyclopedia of Genes and Genomes (KEGG) and Gene Ontology (GO)

Pathway enrichment analyses based on Kyoto Encyclopedia of Genes and Genomes (KEGG) pathways and Gene Ontology (GO) biological processes were conducted using Metascape (https://metascape.org/gp) [37]. The enriched terms were identified based on criteria including an enrichment factor >1.5, a minimum count of 3, and a significance level of *p* < 0.01. Visualization of the top 20 enriched terms was achieved using an online tool (http://www.bioinformatics.com.cn).

### 2.19. Statistical Analysis

Statistical analysis included performing one-way analysis of variance (ANOVA) to assess data significance. Post hoc Tukey's honestly significant difference (HSD) test was conducted to identify specific group differences. A significance level (*α*) of 0.05 was chosen, where differences with a *p* value below this threshold were deemed statistically significant. The results are presented as the means ± standard deviations (SDs) and were analyzed using SPSS version 20.0 statistical software.

## 3. Results and Discussion

### 3.1. Characterizations of Percent Yield and Phenolic and Flavonoid Compound Contents

This study investigated the extraction yields and phenolic and flavonoid compound contents of a Thai herbal recipe containing *Garcinia mangostana* Linn. (*GM*), *Curcuma comosa* Roxb. (*CC*), and *Acanthus ebracteatus* Vahl. (*AE*) using ethanol and water as solvents. The experiment followed a Box‒Behnken design across 15 different ratios of the three herbs, as shown in Table 1. The yields of these herbs and combinations extracted with ethanol varied from 4.67% to 9.17%, with the highest yields observed in formulations with a ratio of 10 : 15 : 5. However, yields of extractions with water were lower overall, ranging from 3.85% to 10.07%, except for formulations with a 10 : 10 : 10 ratio, which had the highest yields. The phenolic contents of the extracts enriched with ethanol were significantly greater, with values of up to 231.67 mg GAE/g, which was particularly evident in the high-*GM* formulations. In contrast, extracts with water had markedly lower phenolic contents, peaking at 37.83 mg GAE/g. Flavonoid contents followed a similar trend, where extracts with ethanol presented higher flavonoid concentrations (up to 143.60 mg QE/g) than did those with water. The observed variations in extraction yields and phytochemical contents can be attributed to solvent polarity and specific herbal matrix interactions. Ethanol, which is a more polar solvent than water, is more effective at extracting phenolic and flavonoid compounds, which are known for their antioxidant activities [38].

**Figure 1:**
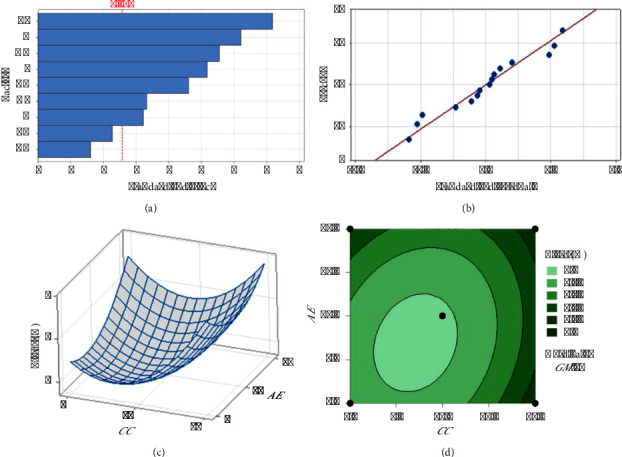
Analysis of the factors affecting yield: (a) Pareto chart of the standardized effect, (b) normal probability plot, (c) 3D surface plot, and (d) contour plot.

**Figure 2:**
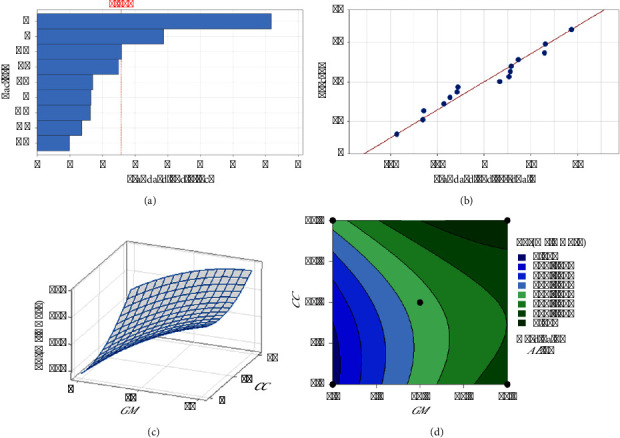
Analysis of factors affecting the phenolic composition: (a) Pareto chart of the standardized effect, (b) normal probability plot, (c) 3D surface plot, and (d) contour plot.

**Figure 3:**
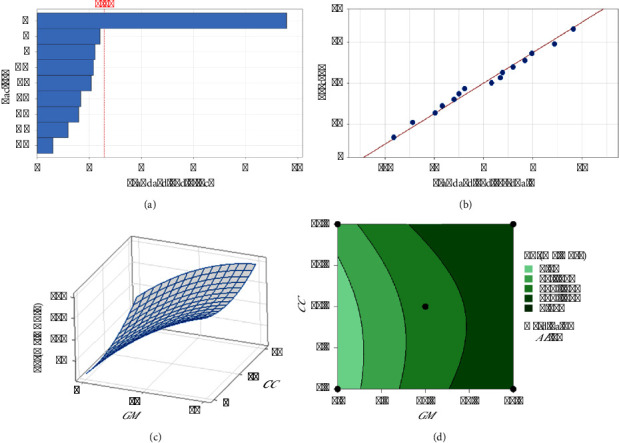
Analysis of factors affecting flavonoid composition: (a) Pareto chart of standardized effects, (b) normal probability plot, (c) 3D surface plot, and (d) contour plot.

The regression models developed from the data (equations (7)–(9)) elucidate the influence of each herbal component on the extraction efficiency and phytochemical content. The standardized effects and interactions between herbal quantities were visually analyzed via Pareto charts (Figures 1(a), 2(a), 3(a)), While normal probability plots (Figures 1(b), 2(b), 3(b)), surface plots (Figures 1(c), 2(c), 3(c)) and contour plots (Figures 1(d), 2(d), 3(d)) further confirmed the model's adequacy and consistency of the experimental results with the theoretical predictions.

(7) $$\begin{array}{c} Y\left(\%\right)=10.38-0.432GM-0.733CC-0.445AE+0.0564\left(GM*GM\right)+0.0730\left(CC*CC\right)+0.0468\left(AE*AE\right)-0.03250\left(GM*CC\right)-0.01570\left(GM*AE\right)-0.02230\left(CC*AE\right), \end{array}$$

(8) $$\begin{array}{c} \text{PE}\left(\text{mgGAE}/g\right)=-18.2+17.42GM-2.80CC+6.49AE-0.628\left(GM*GM\right)+0.962\left(CC*CC\right)-0.380\left(AE*AE\right)-0.504\left(GM*CC\right)+0.958\left(GM*AE\right)-0.632\left(CC*AE\right), \end{array}$$

(9) $$\begin{array}{c} \text{FE}\left(\text{mgQE}/g\right)=-14.0+13.11GM-1.19CC+3.82AE-0.394\left(GM*GM\right)+0.379\left(CC*CC\right)-0.109\left(AE*AE\right)-0.209\left(GM*CC\right)+0.280\left(GM*AE\right)-0.294\left(CC*AE\right). \end{array}$$

The results indicated that after it was extracted with ethanol, *GM* displayed a significant positive effect on both the phenolic and flavonoid contents, likely due to the rich polyphenolic composition of *GM* [39]. *CC* slightly inhibited phenolic and flavonoid extraction, which could be due to its fibrous nature or interaction with other herbs, reducing its solubility [40]. However, *AE* also increased PE and FE, which may be due to its structural properties facilitating solvent penetration and solute leaching [41]. The interaction effects highlighted in equations (8) and (9) indicate that not only individual herb amounts but also their combinations influence the extraction outcomes. For example, the negative interaction between *GM* and *CC* suggests competitive solubility dynamics, whereas positive interactions (e.g., *GM* and *AE* for flavonoids) suggest synergistic effects enhancing solute release.

Figure 1(a) shows the effects of individual herbs and their combinations on yield. “BB” (*CC* alone) had the strongest effect on yield, whereas “AC” (a combination of *GM* and *AE*) had the weakest effect. The numerical threshold at 2.571 likely represents a level of significance, with effects extending beyond this point considered substantial. In Figure 1(b), the plot displays standardized residuals against the predicted values, with the red line indicating perfect agreement between the predicted and actual values (zero residuals), with *R*^2^equal to 97.37. The points closely clustered around the line suggest that the predictive model is quite accurate and reliable for this set of data. In Figure 1(c), the three-dimensional surface plot elucidates the interaction between *CC* (B) and *AE* (C) with respect to their influence on yield. The depicted surface indicates a nonlinear relationship, where the yield is minimized at intermediate levels of both *CC* and *AE*.

Consequently, it is advisable to modulate the levels of *GM*. Integration of these data with Figure 1(a) suggests that an optimal strategy would involve maximizing the concentration of *CC* and minimizing that of *AE* to achieve the highest possible yields, corroborating the observations from Figure 1(d). In summary, to maximize yield, an optimal ratio of *GM* : *CC* : *AE* of 10 : 15 : 5 is recommended on the basis of the analysis. This approach aligns with the empirical data, reinforcing the strategic formulation of component ratios to enhance yield outcomes in practical applications.

In Figure 2(a), the bar for “A” (*GM* alone) indicates the most substantial positive effect on the phenolic content, which was significantly greater than that of all other variables and combinations. These findings suggest that *GM* is a critical factor in increasing the phenolic content. The threshold line at 2.571 likely indicates a level of statistical significance.  Figure 2(b) shows the standardized residuals from a model predicting phenolic content with *R*^2^ equal to 94.80. The data points closely follow the red line, indicating that the model provides an accurate fit to the observed data. In Figure 2(c), the depicted three-dimensional surface plot shows the interaction effects of *GM* and *CC* on the phenolic content. The plot reveals a pronounced upward trajectory in the phenolic content corresponding to increase *GM* levels, which persists irrespective of the *CC* concentration. This pattern indicates that while *CC* positively affects the phenolic content, the influence of *GM* is considerably more pronounced. These data suggest that elevated concentrations of *GM* are associated with the significantly increased phenolic content. Consistent with these findings, Figure 2(d) illustrates that an optimal increase in the phenolic content is achieved when the *GM* concentration is increased to 15 grams, while *CC* is maintained at the lowest 5 grams, and *AE* is held at an intermediate level of 10 grams. This specific combination and ratio optimize the phenolic content, highlighting the critical role of *GM* in the formulation.

Figure 3(a) shows the standardized effects of individual variables (*GM* and *CC*) and their combinations on the flavonoid content. The greatest effect was observed with “A” (*GM* alone), suggesting that *GM* was the most significant factor influencing the flavonoid content. Other combinations and individual variables have lower but still notable effects. The red dashed line at 2.57 likely represents a threshold of statistical significance, indicating that effects beyond this value have a substantial influence on the FE.  Figure 3(b) shows that the points align closely with the red line (ideal fit where residuals = 0) with *R*^2^ equal to 95.97, indicating that the model predictions are highly accurate and reliable, with residuals distributed symmetrically around zero.  Figure 3(c), the three-dimensional plot, displays the interaction between *GM* and *CC* in terms of their influence on the FE. As the *GM* increased, there was a distinct upward trend in the flavonoid content, which persisted irrespective of the *CC* concentration, suggesting a synergistic effect between *GM* and *CC* in enhancing the FE. The shape of the surface implies that the relationship between these variables and FE is nonlinear, with greater gains in flavonoid contents at higher concentrations of both variables. In Figure 3(d), the contour plot provides a two-dimensional view of the same interaction, categorizing different levels of FE concentrations. The darker green areas indicate higher flavonoid contents, particularly at higher levels of *GM* and low levels of *CC*. The plot identifies specific regions where the combination of *GM* and *CC* yields the highest FE, particularly around *GM* levels of 15 and *CC* levels of 5. The analyses underscore *GM* as the primary driver in enhancing flavonoid contents, with its effect being notably potentiated by the presence of a low level of *CC*. This synergy between *GM* and *CC* is crucial for maximizing the FE, suggesting that formulations or treatments aimed at increasing the flavonoid content should consider higher proportions of *GM* and low proportions of *CC*. This result is consistent with the phenolic content results. Therefore, it can be concluded that the optimum proportion for phenolic and maximum flavonoid contents is a *GM* : *CC* : *AE* ratio of 15 : 5:10.

### 3.2. Chemical Compound Analysis via LC-ESI-QTOF-MS/MS

**Figure 4:**
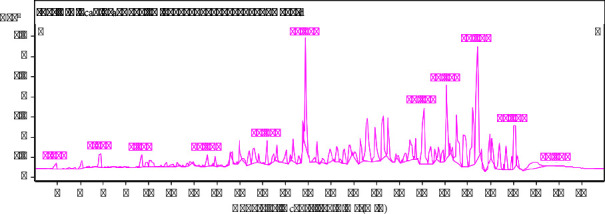
LC-MS/MS peak profile of the herbal combination of *GM* : *CC* : *AE* at a 10 : 15 : 5 ratio.

**Figure 5:**
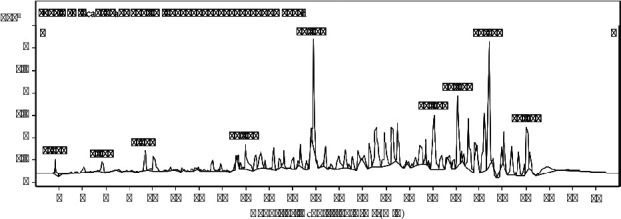
LC-MS/MS peak profile of the herbal combination of *GM* : *CC* : *AE* at a 5 : 10 : 15 ratio.

The LC-MS/MS analysis of the two Thai herbal formulations, *GM* : *CC* : *AE* at ratios of 10 : 15 : 5 and 5 : 10 : 15, revealed a diverse composition of bioactive compounds, including phenolics, flavonoids, terpenes, steroids, and fatty acids. The differences in the peak area percentages and the presence of specific compounds in each formulation are significant, as they may contribute to the overall therapeutic effects of the herbal medicines. In the *GM* : *CC* : *AE* 10 : 15 : 5 formulation as shown in Figure 4, the most prominent compound identified was dimethoxycurcumin, with a peak area of 24.44%. This high concentration suggests that this curcumin derivative might be a key contributor to the anti-inflammatory and antioxidant properties of the formulation. *α*-Mangostin, a well-known xanthone with antioxidant and anti-cancer properties, was also present in a significant amount (14.14%), reinforcing the potential health benefits of this formulation. Other notable compounds included 8-hydroxycarapinic acid (8.59%) and pinolenic acid (1.1%), which are known for their anti-inflammatory properties. The *GM* : *CC* : *AE* 5 : 10 : 15 formulation as shown in Figure 5, on the other hand, exhibited a slightly different profile, with dimethoxycurcumin still being a major component, albeit in a lower concentration (4.54%) compared to the 10 : 15 : 5 formulation. This indicates that while curcumin derivatives remain important, their relative abundance is less in this formulation, possibly altering the therapeutic effects. Rhodomyrtoxin, *α*-mangostin, and mangostinone were also present but at different concentrations compared to the 10 : 15 : 5 formulation. These differences in concentration may influence the formulation's potency and specific health benefits. The presence of various other phenolics, flavonoids, and terpenes in lower concentrations across both formulations suggests that these compounds may work synergistically to enhance the overall efficacy of the herbal medicine. The variation in the chemical composition between the two formulations highlights the importance of ratio adjustments in traditional herbal medicine to achieve desired therapeutic outcomes.

### 3.3. Antioxidants

#### 3.3.1. *In Silico* and *In Vitro* Antioxidants

**Figure 6:**
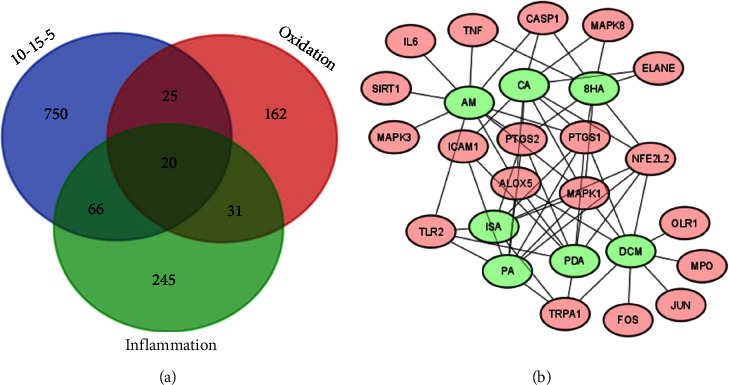
Analysis of the 10 : 15 : 5 herbal combination. (a) Venn diagram showing the overlap of the combination with oxidant and inflammation markers. (b) Network diagram showing the relationships between compounds in the combination and their oxidant and inflammation markers.

The results of the experiment revealed that the *GM* : *CC* : *AE* ratio of 10 : 15 : 5 was the best at resisting free radicals in both DPPH and ABTS, with inhibition abilities as high as 80.33 ± 1.29 and 92.20 ± 0.35%, respectively, at a concentration of 250 *µ*g/mL, as shown in Table 2. Both values were calculated with a fractional percentage inhibitory index (FPI) of 0.5, whereas the other ratios were 0.4, indicating that a *GM* : *CC* : *AE* ratio of 10 : 15 : 5 has an additive effect on antioxidant properties [29]. In addition, the main components of the mentioned proportions were examined, as shown in Figure 4 and Supplementary Table S1. The main components were phenolics, such as 8-hydroxycarapinic acid (*8HA*) at 8.9% and dimethoxycurcumin (*DCM*) at 24.44%. Flavonoids include isovitexin (0.84%) and luteolin (0.64%). The terpenes included rhodomyrtoxin (0.78%) and *α*-mangostin (*AM*) (14.14%). Fatty acids included pinolenic acid (*PA*) (1.1%), isomyristic acid (*ISA*) (1.79%), pentadecylic acid (*PDA*) 2.48%, and other substances, such as sulfamethoxypyridazine (0.82%). As shown in Supplementary Table S4 and Figure 6, most compounds, such as *8HA, CA, DCM, AM, PA, ISA*, and *PDA*, in the formula, approximately 53.64% act by inhibiting free radicals through CCR1, MAPK1, NFKB1, and PTGS1. All 4 types of proteins and enzymes have anti-inflammatory effects. CCR1 (C-C motif chemokine receptor 1) is a chemokine receptor involved in inflammation and the attraction of white blood cells to areas of inflammation; in addition, reports indicate that it is related to antioxidant protection [42]. MAPK1 (mitogen-activated protein kinase 1) is an enzyme involved in intracellular signaling that responds to various factors, such as growth signals and environmental stress. MAPK1 can regulate the expression of genes involved in the response to oxidative stress. Activating MAPK1 may result in cells producing more antioxidants [43]. NFKB1 (nuclear factor kappa-light-chain-enhancer of activated B cells 1) is a protein that regulates genes involved in inflammatory and immune responses. In addition, it has been reported that NFKB1 can control the expression of genes that produce antioxidants such as superoxide dismutase (SOD) and glutathione peroxidase (GPx). Stimulating NFKB1 may increase the production of antioxidants [44]. Prostaglandin-endoperoxide synthase 1 (PTGS1), also known as COX-1, is an enzyme that plays a role in creating prostaglandins, which play a role in the inflammatory process. Inhibiting PTGS1 reduces the production of prostaglandin and oxidative substances that occur in the inflammatory process [45]. In addition, Supplementary Table S4 shows that *8HA, DCM, AM, PA,* and *ISA*, totaling approximately 50.06%, were able to stimulate IMPDH2. Inosine monophosphate dehydrogenase 2 (IMPDH2) is an enzyme that plays an important role in the synthesis of nucleic acids by converting inosine monophosphate (IMP) to xanthosine monophosphate (XMP), which is an important step in the synthesis of purine nucleotides. IMPDH2 expression may affect the production of antioxidant substances in cells. The study in [46] has shown that IMPDH2 induction can help fight free radicals, so it is possible that the main compounds in the *GM* : *CC* : *AE* herbal formula at a ratio of 10 : 15 : 5 are *8HA, CA, DCM, AM, PA, ISA,* and *PDA*; these play important roles in antioxidant protection through CCR1, MAPK1, NFKB1, PTGS1, and IMPDH2 induction.

In addition, if we consider the important substances that affect the fight against free radicals and inflammation, there are a total of 20 types of proteins and enzymes, as shown in Figure 6(a) and Supplementary Table S3, and compounds that affect the antioxidant proteins and enzymes. The proteins associated with the highest levels of free radicals and inflammation are *CA* and *AM*, which affect 10 different proteins and enzymes: *CA* affects the proteins and enzymes XDH, ALOX5, ELANE, NFE2L2, CASP1, PTGS2, MAPK1, MAPK8, ICAM1, and PTGS1, and *AM* affects the proteins and enzymes ALOX5, TNF, CASP1, PTGS2, IL6, MAPK1, MAPK3, SIRT1, PTGS1, and TLR2, as shown in Figure 6(b) and Supplementary Table S5. Therefore, the main active antioxidant and anti-inflammatory ingredients are chlorogenic acid and *α*-mangostin.

**Figure 7:**
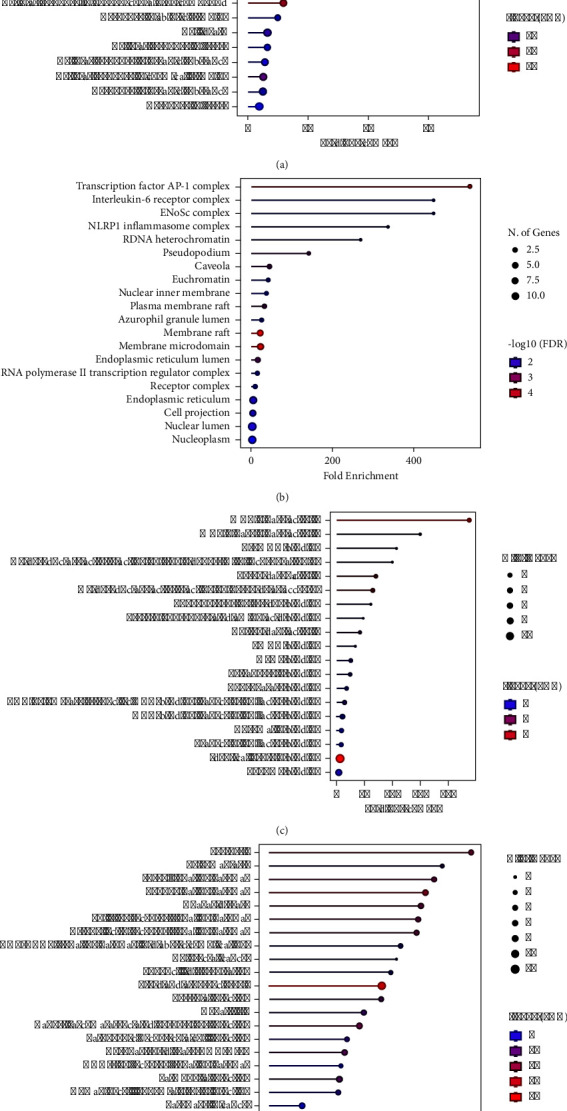
Gene Ontology (GO) enrichment analysis of the herbal combination 10 : 15 : 5 containing (a) biological process, (b) cellular component, (c) molecular function, and (d) KEGG pathway terms.

A total of 20 genes were analyzed via Gene Ontology (GO). The results showed that the recipe of *GM* : *CC* : *AE* at a ratio of 10 : 15 : 5 provided enrichment plots, as shown in Figure 7. These plots indicate the biological pathways and functions that are significantly enriched in antioxidant activity. As shown in Figure 7(a), the cellular response to reactive oxygen species (ROS) was significantly enriched, indicating a strong antioxidant response to oxidative stress [47]. The high fold enrichment and low FDR values suggest that the recipe components significantly influence this pathway. The cellular response to metal ions and chemical stress, which is closely related to antioxidant defense mechanisms, is also highly enriched [48]. Responses to external stimuli, such as stress, lipopolysaccharides, and biotic stimuli, are enriched, further indicating the potential of the recipe in modulating cellular responses to oxidative stress [49]. As shown in Figure 7(b), the enriched cellular components of the transcription factor AP-1 complex and the interleukin-6 receptor complex were highly enriched. AP-1 is known for its role in regulating gene expression in response to oxidative stress [50]. Cytoplasmic components, such as the inflammasome complex and heterochromatin, are involved in cellular stress responses [51]. As shown in Figure 7(c), the enriched molecular functions revealed that MAP kinase activity, oxidoreductase activity, and antioxidant activity were significantly enriched. These functions are directly related to the mitigation of oxidative damage and ROS detoxification [52]. Enzyme-binding and DNA-binding transcription factor activity suggest roles in regulating antioxidant gene expression [53]. As shown in Figure 7(d), the enriched KEGG pathways revealed that the pertussis and IL-17 signaling pathways were enriched, indicating potential immune-modulatory effects [54]. The toll-like receptor and AGE-RAGE signaling pathways, which are involved in the response to oxidative stress [55], are significantly enriched. Various cancer pathways and virus infection pathways are also enriched, suggesting broader implications of the recipe in managing oxidative stress-related diseases [56].

The enrichment plots indicate that the Thai recipe containing a *GM* : *CC* : *AE* ratio of 10 : 15 : 5 has strong potential for enhancing cellular responses to oxidative stress, modulating key signaling pathways involved in the immune response and inflammation, influencing antioxidant enzyme activities and related transcriptional activities, and providing broader protective effects against oxidative stress-related conditions such as inflammation and infections. Therefore, the components of the Thai recipe work synergistically to bolster the body's antioxidant defenses, potentially offering therapeutic benefits in managing oxidative stress and related diseases.

#### 3.3.2. Combination Index Analysis and Optimal Ratio of Antioxidants

The antioxidant activity was investigated via the DPPH and ABTS methods to determine the appropriate amount of antioxidant activity. The experimental results revealed that the antioxidant activity of the preparation at 125 *µ*g/mL could exert additive and synergistic effects on DPPH and ABTS, respectively.

**Figure 8:**
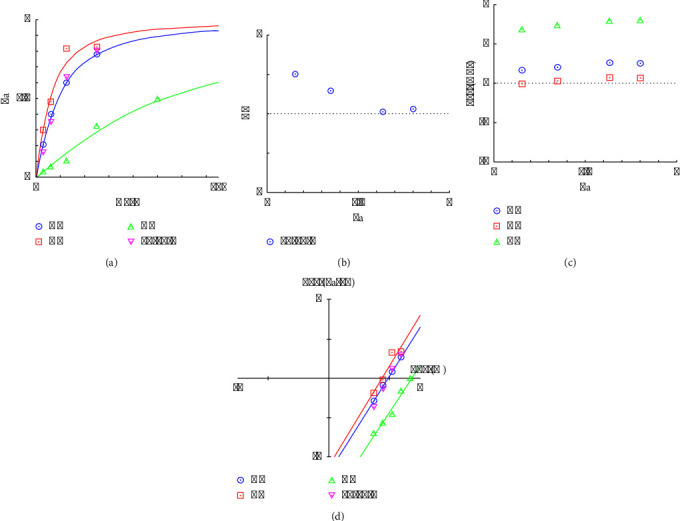
Antioxidant activity analysis of the 10 : 15 : 5 herbal combination via a DPPH assay containing (a) dose-response curves, (b) the combination index (CI), (c) the dose-reduction index (DRI), and (d) isobologram analysis.

**Figure 9:**
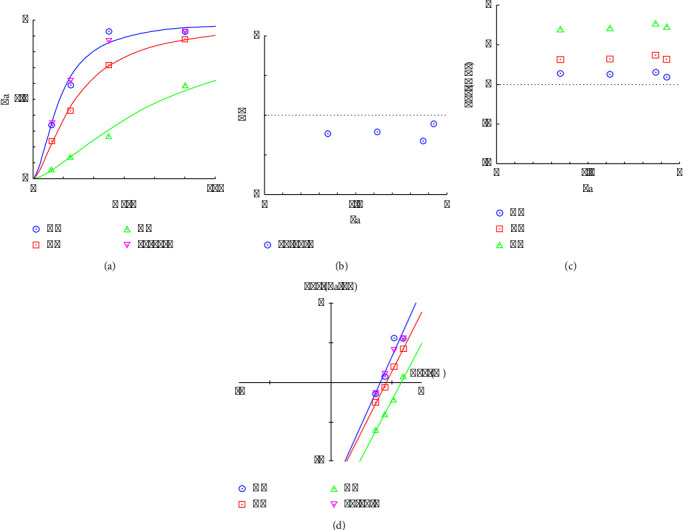
Antioxidant activity analysis of the 10 : 15 : 5 herbal combination via the ABTS assay: (a) dose-response curves, (b) the combination index (CI), (c) the dose-reduction index (DRI), and (d) isobologram analysis.

In Figure 8(a), the *y*-axis (Fa) represents the antioxidant affected by the dose, whereas the *x*-axis represents the dose. Compared with *GM* (blue) and *AE* (green), *CC* (red) has the highest efficacy, achieving a greater Fa at lower doses. The combination (M-H-L, purple) closely followed the curve for *GM*, suggesting that the medium dose of *GM* dominated the DPPH antioxidant effect of this combination. As shown in Figure 9(a), *CC* (blue) and *GM* (red) presented pronounced dose-dependent increases in activity, with higher activities at elevated doses. *AE* (green) displays a more gradual increase. The profile of the combination (M-H-L, purple) closely resembled that of *CC*, suggesting that the high dose of *CC* may be primarily responsible for the observed ABTS antioxidant activity in the combination. In Figure 8(b), the CI values for the combination are plotted against the fraction affected. A CI value less than 1 indicates synergism, a CI value equal to 1 indicates an additive effect, and a CI value greater than 1 indicates antagonism. Here, the CI values for the combination are approximately 1 when the dose is more than 125 *µ*g/mL, suggesting that the combination effect is roughly additive across the range of effects studied. As shown in Figure 9(b), the CI values are all less than 1 across the fractions affected, suggesting that the combination of these herbs has a synergistic effect at 31.25 *µ*g/mL, increasing the antioxidant activity more than each herb alone. In Figures 8(c) and 9(c), DRI values indicate how much the dose of each component in combination can be reduced while still achieving the same effect as when used alone. DRI values greater than 1 suggest a beneficial interaction [57]. In this study, all the herbs presented DRIs of approximately 1 or slightly greater than 1, suggesting slight beneficial interactions or nearly equivalent dosing efficiencies in combination. As shown in Figures 8(d) and 9(d), this plot generally helps determine the potency and efficacy of drugs. The lines for all individual herbs and the combination are relatively close, indicating similar potency levels. Compared with individual herbs, the combination does not appear to drastically enhance or reduce the overall effect. In particular, the lines for all the herbs and the combination were relatively similar, with *GM, CC*, and the combination showing similar potencies. *AE* shows a slightly different slope, suggesting a different potency or efficacy profile.

**Figure 10:**
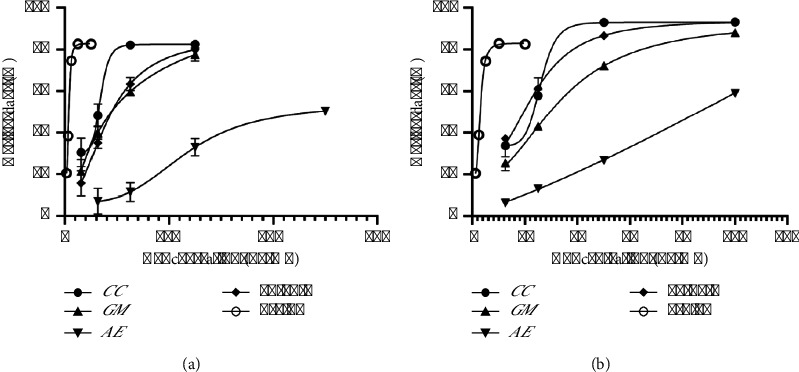
Antioxidant activity of the herbal combination 10 : 15 : 5 and individual herbs determined via the (a) DPPH assay and (b) ABTS assay.

Moreover, as shown in Figures 10(a) and 10(b), the amounts that could inhibit half of the free radicals of the *GM* : *CC* : *AE* formulation at a ratio of 10 : 15 : 5 were 82.97 ± 8.30 and 23.94 ± 4.34 *µ*g/mL for DPPH and ABTS, respectively. The DPPH results revealed that the herbal ingredients *GM, CC,* and *AE* had values of 101.72 ± 77.36, 67.64 ± 12.01, and 232.7 ± 49.2 *µ*g/mL, respectively. The inhibitory values of the preparations were in the range of herbs. The results of the analysis were in the same direction, i.e., additive effects, as shown in Figure 8(b), whereas the ABTS results revealed that the values of the *GM*, *CC*, and *AE* herbs were 33.37 ± 2.24, 25.95 ± 0.83, and 86.43 ± 0.54 *µ*g/mL, respectively, in which the inhibitory effect of the preparation was greater than that of the herbal medicine. The results of the analysis were similar, i.e., there was a synergistic effect, as shown in Figure 9(b). However, the inhibition values of both DPPH and ABTS were still lower than those of Trolox, with IC_50_ values of 7.70 ± 0.77 and 3.08 ± 0.07 *µ*g/mL, respectively. Although the inhibitory effect of this formulation is less effective than that of Trolox, the use of a variety of herbs can provide additional therapeutic effects, such as anti-inflammatory effects, and can reduce the toxicity of using only one herb.

Therefore, the data suggest that the combination of *CC, GM,* and *AE* at the specified doses (medium *GM*, high *CC*, and low *AE*) does not result in a strong synergistic effect on the antioxidant DPPH but rather an additive interaction where the effects of the individual herbs are not significantly enhanced or reduced when in combination. This approach is useful in clinical settings where the goal is to achieve the desired therapeutic effects without increasing side effects. However, the ABTS assay results suggest that the combination of *CC, GM,* and *AE*, particularly with a high dose of *CC*, synergistically enhances antioxidant activity compared with the individual herbs. This finding indicates the potential for reducing the dosage of each herb in combination while achieving desirable antioxidant effects, and it is safer than using only one herb.

### 3.4. Anti-Inflammatory Effects

#### 3.4.1. Combination Index Analysis

As shown in Table 3, ethanol extraction generally has greater anti-inflammatory activity than water extraction for the same herbal combinations. Among the combinations tested, the combination of 10 g of *GM*, 10 g of *CC*, and 10 g of *AE* had the highest anti-inflammatory activity (64.74 ± 4.86%) in aqueous extraction. These findings suggest that a balanced proportion of the three herbs is optimal for aqueous extraction to achieve significant anti-inflammatory effects. However, other combinations resulted in lower anti-inflammatory percentages, indicating that the proportions of the herbs significantly affect the efficacy of the extract. In ethanol extraction, the highest anti-inflammatory activity was observed with the combination of 5 g of *GM*, 10 g of *CC*, and 15 g of *AE* (98.86 ± 1.12%), closely followed by 10 g of *GM*, 10 g of *CC*, and 10 g of *AE* (98.29 ± 0.93%). These results suggest that ethanol extraction is particularly effective in enhancing the anti-inflammatory properties of the herbal mixtures because the compounds that act as inhibitors are mainly phenolics, such as pseudolaric acid B (1.36%), dimethoxycurcumin (4.54%), 8-hydroxycarapinic acid (1.02%), mangostinone (1.07%), garcinone C (2.3%), and mangostenol (1.02%), as shown in Figure 5 and Supplementary Table S2, which have the ability to dissolve ethanol better than water extraction does [58]. This study revealed that both the proportion of herbs and the extraction method significantly influence the anti-inflammatory efficacy. In particular, ethanol extraction seems to be more effective in maximizing the anti-inflammatory properties of the tested combinations. The combination of 5 g of *GM*, 10 g of *CC*, and 15 g of *AE* with ethanol extraction had the highest potential for anti-inflammatory activity.

**Figure 11:**
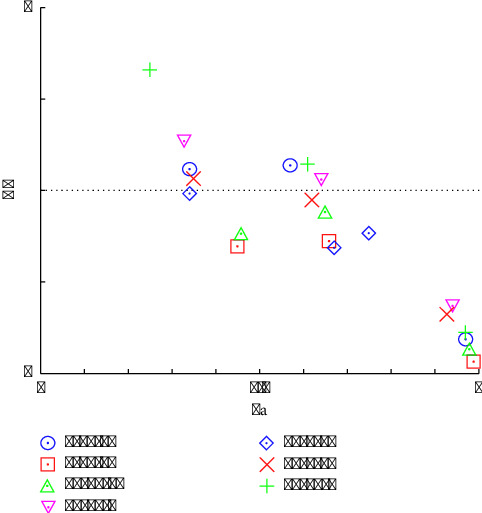
Anti-inflammatory activity analysis of various herbal combinations in terms of combination index (CI) plots.

Figure 11 shows the CI values (*y*-axis) plotted against the fraction affected (Fa, *x*-axis) for different herbal combinations. The recipes 5-15-10 (circle), 10-15-5 (inverted triangle), 15-10-5 (plus), and 15-5-10 (cross) show less potential to inhibit inflammation because these combinations have CI values greater than 1, indicating an antagonistic effect at low concentrations. These findings suggest that the combined effect of these herb ratios is less effective than the sum of their individual effects. The ratio of 10 : 5 : 15 (diamonds) shows medium potential to inhibit inflammation because this combination has CI values of approximately 1, reflecting an additive effect, where the combined anti-inflammatory activity is equivalent to the sum of the individual effects of the herbs. Moreover, 5-10-15 (squares) and 10-10-10 (triangles) show synergistic effects. Both combinations have CI values less than 1, indicating a synergistic effect. However, the combination of 5-10-15 (squares) shows a more pronounced synergistic efficiency than 10-10-10 (triangles), as evidenced by its position being lower on the graph. This finding indicates a stronger combined anti-inflammatory activity for the 5-10-15 ratio. The analysis confirmed that specific combinations of *Garcinia mangostana* Linn., *Curcuma comosa* Roxb., and *Acanthus ebracteatus* Vahl. can either enhance or inhibit anti-inflammatory effects depending on their ratio. The combination of 5 g of *GM*, 10 g of *CC*, and 15 g of *AE* (5-10-15) demonstrated superior synergistic efficiency, outperforming the 10-10-10 combination. This finding highlights the importance of optimizing herbal proportions to achieve maximum therapeutic benefits through synergistic interactions.

#### 3.4.2. *In Silico* Anti-Inflammatory Effects

**Figure 12:**
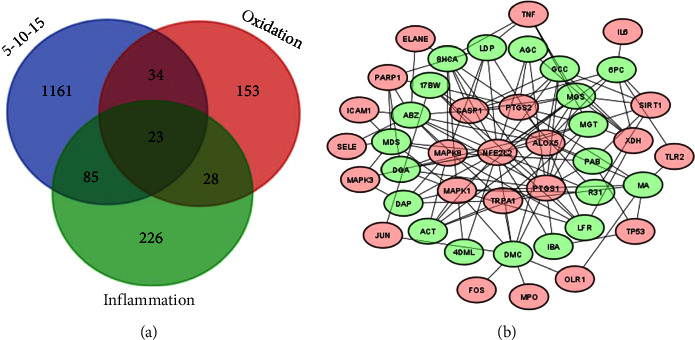
Analysis of the 5 : 10 : 15 herbal combination. (a) Venn diagram showing the overlap of the combination with oxidants and inflammation markers. (b) Network diagram demonstrating the relationships between the compounds in the combination and their oxidants and inflammation markers.

The proportion that yields the optimal synergistic effect is the ratio of 5 : 10 : 15, which effectively inhibits inflammation and functions as both an antioxidant agent and an anti-inflammatory agent. A total of 23 genes or proteins were found to be involved, as depicted in Figure 12(a). These include XDH, ALOX5, ELANE, SELE, NFE2L2, TNF, MPO, CASP1, TRPA1, PTGS2, OLR1, IL6, PARP1, MAPK1, FOS, MAPK8, ICAM1, MAPK3, TP53, SIRT1, PTGS1, TLR2, and JUN, as shown in Supplementary Table S6. The examination further revealed that the NFE2L2 gene serves as the primary target protein influenced by all 19 substances. These substances include PAB, MGS, GCC, ABZ, LFR, AGC, R31, 6PC, DAP, MDS, LDP, IBA, 17BW, DML, DGA, MA, DMC, 8HCA, MGT, and ACT, all of which act upon the NFE2L2 protein, as shown in Supplementary Table S7 and Figure 12(b).

NFE2L2 is recognized for its role in controlling the expression of several genes involved in antioxidant and cytoprotective functions. Upon activation, NFE2L2 migrates to the nucleus and interacts with the antioxidant response element (ARE) in specific gene promoter regions. This interaction results in the activation of these genes at the transcriptional level. This process leads to the generation of several antioxidant enzymes and proteins that aid in reducing oxidative stress and inflammation [59]. NFE2L2 enhances the expression of antioxidant proteins, including heme oxygenase-1 (HO-1), NAD(P) oxidoreductase 1 (NQO1), and glutathione S-transferases (GSTs). These proteins decrease the amount of reactive oxygen species (ROS), hence reducing oxidative stress and preventing the activation of inflammatory pathways [60]. The activation of NFE2L2 inhibits the production of proinflammatory cytokines such as TNF-*α*, IL-6, and IL-1*β*. The inhibition of this process occurs by reducing the activity of nuclear factor-kappa B (NF-*κ*B), a crucial controller of inflammatory reactions [61].

In summary, the 5-10-15 combination has anti-inflammatory benefits by decreasing oxidative stress through increased expression of NFE2L2. This increase suppresses the activity of proinflammatory cytokines and regulates the pathways involved in inflammation. Therefore, the synergistic effect of the 5-10-15 combination increases the expression of NFE2L2, making it a crucial target for therapeutic approaches that aim to reduce inflammation and damage caused by oxidative stress.

**Figure 13:**
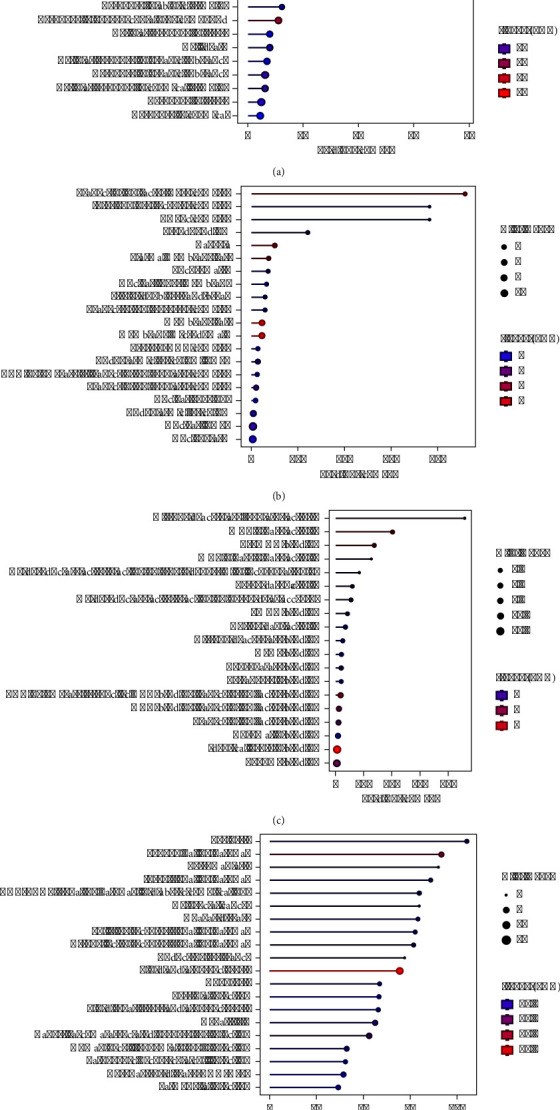
Gene Ontology (GO) enrichment analysis of the herbal combination 5 : 10 : 15, including (a) biological process, (b) cellular component, (c) molecular function, and (d) KEGG pathway terms.

Figure 13(a) illustrates the cellular reactions to many types of stress, specifically reactive oxygen species, oxidative stress, and chemical stress. The high fold enrichment and significant number of genes involved suggest a robust cellular response to inflammation and oxidative damage. Key genes involved in NFE2L2 (NRF2) are central to the antioxidant response, regulating the expression of antioxidant proteins. TNF and IL6 are proinflammatory cytokines that are critical in the inflammatory response. CASP1 is involved in the activation of proinflammatory cytokines. MAPK1, MAPK3, and MAPK8 are part of the MAP kinase signaling pathway, which is essential in inflammatory responses [62].  Figure 13(b) shows the enrichment of various cellular components, such as transcription factor complexes and membrane components. These elements are crucial for the regulation and propagation of inflammatory signals. Notably, NFE2L2 is involved in transcription factor complexes, indicating its role in regulating genes that mitigate oxidative stress and inflammation.  Figure 13(c) shows the molecular functions of the identified genes, such as histone deacetylase activity, MAP kinase activity, and oxidoreductase activity. NFE2L2 and SIRT1 are key players in antioxidant defense and inflammatory response modulation [63]. Histone deacetylase activity is important for regulating gene expression in response to inflammation [64]. Oxidoreductase activity involves enzymes that play roles in the oxidative stress response via detoxification in the liver [65].  Figure 13(d) shows the involvement of these genes in various signaling pathways, including the TNF signaling, IL-17 signaling, and AGE-RAGE signaling pathways, all of which are critical in mediating inflammatory responses. The TNF signaling pathway is a major pathway involved in inflammation. The MAPK signaling pathway is crucial for transmitting inflammatory signals. Furthermore, the AGE-RAGE signaling pathway is involved in chronic inflammation and oxidative stress [66].

The synergistic effects of *Garcinia mangostana* Linn., *Curcuma comosa* Roxb., and *Acanthus ebracteatus* Vahl. appear to efficiently suppress inflammatory pathways. This is supported by the notable increase in the expression of genes involved in the TNF and IL-17 signaling pathways, as well as the involvement of transcription factors such as NFE2L2. NFE2L2 (NRF2) is notable for its role in controlling the activity of genes that are responsible for antioxidant defense, leading to a decrease in inflammation caused by oxidative stress. Furthermore, the notable increase in oxidoreductase activity and the participation of genes such as NFE2L2 and SIRT1, and several MAP kinases suggest a robust antioxidant response. NFE2L2 functions as a primary controller of the antioxidant response, amplifying the production of various antioxidant proteins that alleviate the cellular harm caused by oxidative stress. Hence, the combination of *Garcinia mangostana* Linn., *Curcuma comosa* Roxb., and *Acanthus ebracteatus* Vahl. (at a ratio of 5 : 10 : 15) has strong anti-inflammatory and antioxidant characteristics. The participation of 23 crucial genes and their notable enrichment in pertinent pathways underscore the efficacy of this combination in controlling inflammation and oxidative stress, with NFE2L2 playing a vital role in these mechanisms.

#### 3.4.3. *In Vitro* Anti-Inflammatory Effects and Combination Analysis of the Optimal Ratio

**Figure 14:**
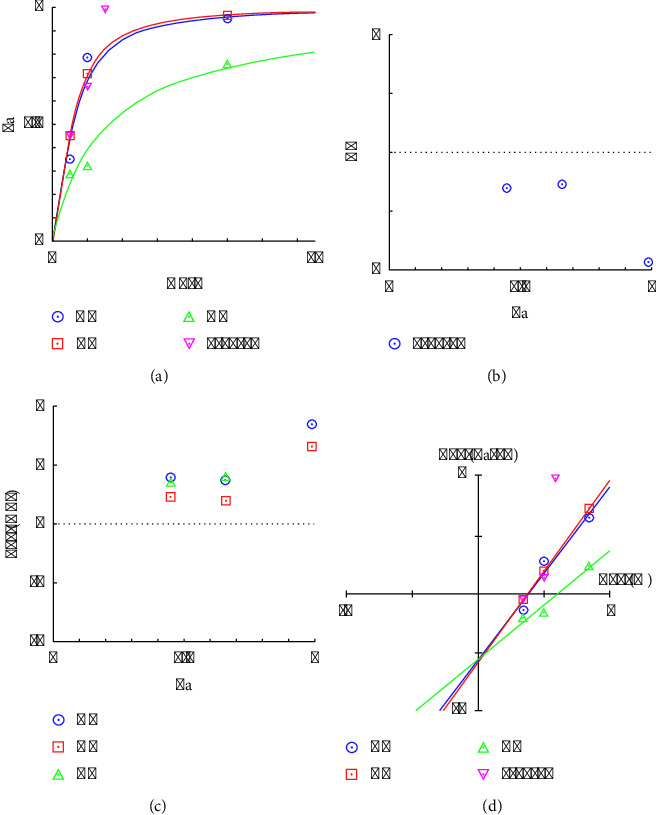
Anti-inflammatory activity analysis of the 5 : 10 : 15 herbal combination, including (a) dose-response curves, (b) combination indices (CIs), (c) dose-reduction indices (DRIs), and (d) isobologram analysis.

In Figure 14(a), the combination curve (reverse triangle symbols) generally lies above the individual herb curves, especially at lower doses, suggesting that the combination is more effective or requires a lower dose to achieve efficacy levels similar to those of the individual herbs. In Figure 14(b), the CI values appear to be around or below 1 (particularly at higher Fa levels), indicating that the combination tends to be synergistic, especially at higher efficacy levels. In Figure 14(c), combinations of *GM, CC,* and *AE* are more effective together than individually, especially for *GM* and *AE*, as indicated by their high DRI values at higher efficacy levels. Points above the zero line (log (DRI) > 0) indicate a synergistic interaction, allowing for dose reduction. *GM* and *AE* show positive log (DRI) values across different Fa values, indicating effective dose reduction through synergy. *CC* has values closer to zero, suggesting less pronounced synergy. As shown in Figure 14(d), the lines for all the herbs and the combination were relatively close to those of *GM* and *CC* except for the high-dose combination, which was more potent. Therefore, the combination of *GM, CC,* and *AE* at the ratio of 5 : 10 : 15 has synergistic effects, which is particularly evident from the dose-response and CI graphs. Compared with individual components, the combination is more effective at lower doses and maintains synergy across a range of efficacies (Fa). This synergy could be particularly beneficial for anti-inflammatory activity, where reducing the dosage while maintaining or enhancing efficacy can minimize side effects and improve patient outcomes.

**Figure 15:**
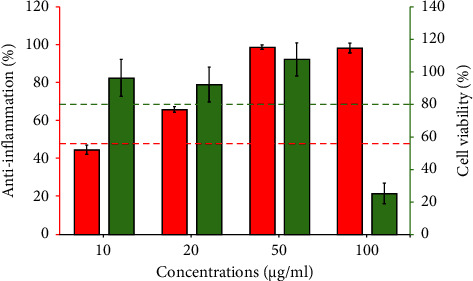
Anti-inflammatory activity and cytotoxicity of the herbal combination 5 : 10 : 15 on Raw 264.7 cells.

Figure 15 presents the anti-inflammatory properties of a recipe containing *Garcinia mangostana* Linn., *Curcuma comosa* Roxb., and *Acanthus ebracteatus* Vahl. at concentrations of 5, 10, and 15, respectively. The green bars refer to the viability of RAW 264.7 cells, while the red bars refer to the anti-inflammatory effect measured by nitric oxide inhibition. The recipe was chosen because it is optimized for these effects. The green bars represent the proportion of viable RAW 264.7 cells, with a viability threshold of 80% considered indicative of cell safety [67]. At concentrations of 10, 20, and 50, the cell viability was greater than 80%. The green dashed line represents 80% cell viability, indicating that cell viability was maintained at an acceptable level for all tested concentrations. The red bars show the percentage of anti-inflammatory effects through nitric oxide inhibition. At concentrations of 20, 50, and 100, the anti-inflammatory effect was greater than 50%. The red dashed line represents 50% inhibition, indicating the baseline for a significant anti-inflammatory effect, which was exceeded at all the tested concentrations. In summary, although a concentration of 10 *µ*g/mL is safe for RAW 264.7 cells, its anti-inflammatory efficacy is less than half. Moreover, at 100 *µ*g/mL, the anti-inflammatory effect was strong, but it significantly impacted the viability of the RAW 264.7 cells. Therefore, the optimal concentration range for anti-inflammatory activity is 20–50 *µ*g/mL. This formulation is effective in maintaining nontoxic cell viability while providing strong anti-inflammatory effects.

### 3.5. Cytotoxicity Analysis

**Figure 16:**
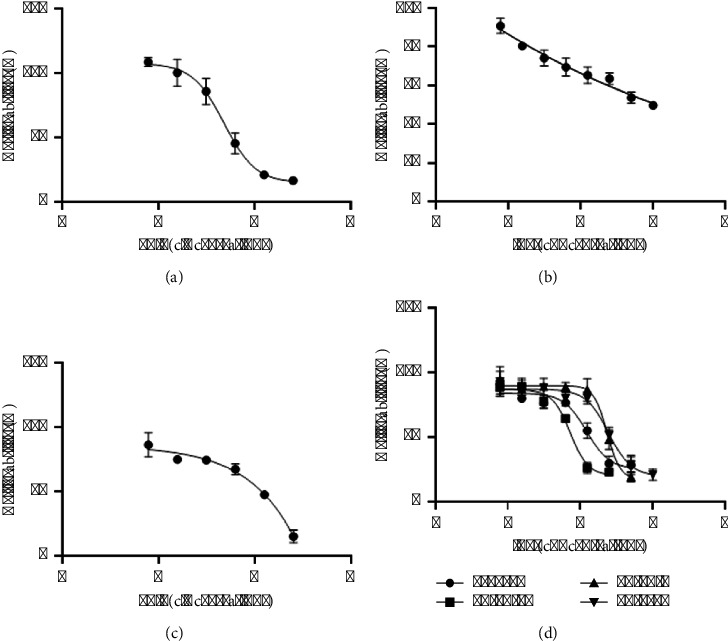
Cytotoxicity of L929 fibroblasts to (a) the *Garcinia mangostana* extract, (b) the *Acanthus ebracteatus* extract, (c) the *Curcuma comosa* extract, and (d) the Thai herbal recipe containing the *Garcinia mangostana*: *Curcuma comosa*: *Acanthus ebracteatus* extract.

The study also included a cytotoxicity assessment using L929 fibroblasts to evaluate the safety profile of the herbal extracts. The cell viability value that indicates safety to cells is 80%, which indicates the ability to inhibit cell growth by 20% (IC_20_) [23, 68]. The results indicated varying degrees of cytotoxicity among the different herbal combinations and extraction methods, with *Garcinia mangostana* showing the least cytotoxic effects, followed by *Acanthus ebracteatus* and *Curcuma comosa*, which presented the highest levels of cytotoxicity. As shown in Figure 16(a), among the individual herbs, *Garcinia mangostana* had the highest safety margin, with an IC_20_ value of 35.21 *µ*g/mL. This higher IC_20_ value suggests that *Garcinia mangostana* can be used at the highest concentrations to maintain cell viability, supporting its potential for safe therapeutic applications. As shown in Figure 16(b), *Acanthus ebracteatus* presented an IC_20_ value of 16.19 *µ*g/mL. This lower IC_20_ value suggests that it can be used at lower concentrations than can *Garcinia mangostana* for maintaining cell viability. As shown in Figure 16(c), *Curcuma comosa* has an IC_20_ value of 10.68 *µ*g/mL, indicating the highest sensitivity of cells to this herb. This lowest threshold requires careful dosage management to avoid cytotoxic effects. As shown in Figures 16(d), 5, 10, and 15, 35.93 ± 23.29 *µ*g/mL showed cytotoxicity similar to that of *GM* (35.21 *µ*g/mL), indicating a balanced effect among the components that did not overly exacerbate cell toxicity. However, 10-10-10 (27.43 ± 8.59 *µ*g/mL) was more cytotoxic than the 5-10-15 combination but less than *GM* alone, suggesting a moderate increase in cytotoxic effects, possibly due to the presence of *GM* in equal proportions with the other extracts. The 5-10-15 and 10-10-10 combinations appear most effective, as shown in Table 4. In particular, 5-10-15 stands out for its high efficacy across all the tested concentrations and its low toxicity, indicating that it could be an optimal choice for further development in anti-inflammatory treatments. The 10-15-5 and 15-5-10 combinations had relatively high IC_20_ values (147.04 and 128.16 *µ*g/mL, respectively), suggesting increased safety and tolerance in the cellular models, as shown in Figure 16(d) as triangle and reverse triangle shapes, respectively. These ratios appear to mitigate the cytotoxic effects more effectively, indicating beneficial interactions among the herbs that increase cell survival. As shown in Figure 10, the formulation with the best free radical inhibition efficiency was *GM* : *CC* : *AE* at 10-15-5. The IC_50_ concentrations of 82.97 ± 8.30 *µ*g/mL and 23.94 ± 4.34 *µ*g/mL from the DPPH and ABTS assays, respectively, are safe; therefore, the appropriate concentration for antioxidant use is 82.97–147.04 *µ*g/mL, indicating that this concentration could be an optimal choice for further development in antioxidant applications.

The analysis of IC_20_ values offers significant insights into the safety profiles of these herbal combinations. It is evident that certain combinations, particularly those with higher IC_20_ values, are likely to be safer for therapeutic use. Compared with individual herb components, these combinations reduce the cytotoxic effects.

### 3.6. Interrelationship between Oxidative Stress and Inflammation in Polyherbal Formulations

In this study, we identified two effective ratios, 10 : 15 : 5 and 5 : 10 : 15, for antioxidant and anti-inflammatory effects. However, these results should be contextualized within the broader understanding of the intricate relationship between oxidative stress and inflammation. Oxidative stress and inflammation are closely linked, with each process potentially exacerbating the other. For example, oxidative stress can trigger inflammatory responses by activating various signaling pathways, including the NF-*κ*B pathway, leading to the production of proinflammatory cytokines [69]. Conversely, inflammation can further increase oxidative stress by increasing the production of reactive oxygen species (ROS), thus creating a vicious cycle that exacerbates tissue damage [70]. The ratios observed in our study likely reflect the complex interactions between these two processes. The different optimal ratios for antioxidant and anti-inflammatory effects suggest that the balance between oxidative stress and inflammation may require precise modulation to achieve maximal therapeutic efficacy. This finding is particularly relevant given the evidence that antioxidants can mitigate inflammation by neutralizing ROS, thereby reducing the activation of proinflammatory pathways. For example, in the study by Jedli et al. [69], *Zingiber officinale* extract demonstrated significant protective effects against lung oxidative injury and inflammation in an asthma model, emphasizing the importance of targeting both oxidative stress and inflammation simultaneously. In light of this, future studies could explore the potential for identifying a single, optimal ratio that effectively balances both antioxidant and anti-inflammatory effects. In addition, understanding the molecular mechanisms underlying these interactions could provide deeper insights into how to best combine these botanicals for therapeutic purposes.

### 3.7. Selectivity Index for Anti-Inflammatory Studies

The selectivity index (SI) was calculated to evaluate the balance between anti-inflammatory efficacy and cytotoxicity across various herbal formulations and single-component extracts. The SI, which compares the IC_50_ values for anti-inflammatory activity in Raw 264.7 cells with the IC_20_ values for cytotoxicity in L929 fibroblasts, serves as a key indicator of therapeutic selectivity and safety. Single-component extracts of *Curcuma comosa* (*CC*) and *Acanthus ebracteatus* (*AE*) presented low SI values of 0.79 and 0.32, respectively, indicating a high degree of cytotoxic risk relative to their anti-inflammatory benefits. In contrast, *Garcinia mangostana* (*GM*) alone presented a much more favorable SI of 3.03, which is comparable to that of the 5 : 10 : 15 formulation. These findings suggest that *GM*, even as a single-component extract, offers a good balance between efficacy and safety, making it a strong candidate for use in anti-inflammatory applications. The combination at a 5 : 10 : 15 ratio had an SI of 2.98, closely mirroring the selectivity of GM alone. This formulation is nearly three times more effective in targeting inflammation while minimizing cytotoxicity, highlighting its potential as a well-balanced therapeutic option. The ratio of 10 : 10 : 10 resulted in a slightly lower IC_50_ value of 11.47 *µ*g/mL, indicating stronger anti-inflammatory activity than the 5 : 10 : 15 formulation. However, its SI of 2.39, although still within an acceptable range, suggests a narrower safety margin. This finding indicates that while the 10 : 10 : 10 ratio is more potent in reducing inflammation, it is less selective, leading to a greater potential for cytotoxicity in fibroblasts. Overall, these findings underscore the importance of considering both anti-inflammatory potency and safety in therapeutic development. Owing to its optimal combination of efficacy and safety, the 5 : 10 : 15 ratio is a superior candidate for further development of anti-inflammatory therapies. The similar SI value for *GM* alone further suggests that it is a key contributor to the overall effectiveness and safety of the formulation.

### 3.8. Limitations of the Study

One of the key limitations of this study is the observation that the optimal ratios for antioxidant and anti-inflammatory effects are not the same. The study identified different ratios for maximizing these effects: the 10 : 15 : 5 ratio was found to be most effective for increasing antioxidant activity, whereas the 5 : 10 : 15 ratio was optimal for exerting anti-inflammatory effects. This finding indicates that a single, unified ratio that maximizes both bioactivities could not be identified within the scope of this study. However, it is important to note that the use of multiple herbs in combination, as explored in this study, offers significant advantages over the use of single herbs. The diversity in bioactive compounds from different herbs allows for a broader therapeutic spectrum, potentially addressing multiple pathways involved in disease processes. This means that even if a single optimal ratio for both antioxidant and anti-inflammatory effects could not be determined, the combination of herbs still provides a more comprehensive approach to treatment, targeting different aspects of inflammation and oxidative stress more effectively than single-component therapies could. Therefore, this limitation does not diminish the therapeutic potential of the formulations but rather highlights the complexity and the need for further research to explore whether a balanced ratio that effectively combines both activities can be achieved. Alternatively, separate formulations might be required depending on the desired therapeutic effect. This limitation should be taken into consideration when interpreting the results and planning future studies.

## 4. Conclusion

The *Garcinia mangostana*, *Curcuma comosa* Roxb., and *Acanthus ebracteatus* optimal combinations have significant potential as antioxidants and anti-inflammatory agents. For antioxidant purposes, the most effective ratio was 10 : 15 : 5, demonstrating notable inhibition, with IC_50_ values of 82.97 ± 8.30 *µ*g/mL in the DPPH assays and 23.94 ± 4.34 *µ*g/mL in the ABTS assays. This antioxidant activity primarily targets the transcription factor AP-1 complex, which is crucial for regulating gene expression in response to oxidative stress. The synergistic effect of this ratio becomes apparent at concentrations above 31.25 *µ*g/mL for ABTS activity and over 125 *µ*g/mL for DPPH activity, with a safe dose established at 147.04 *µ*g/mL. For anti-inflammatory effects, the best ratio was 5 : 10 : 15, with an IC_50_ value of 12.05 ± 0.94 *µ*g/mL. This mechanism involves the upregulation of NFE2L2, a key factor in combating inflammation. The synergistic anti-inflammatory activity was evident at concentrations starting at 10 *µ*g/mL, with a safe dose determined to be 35.93 ± 23.29 *µ*g/mL. These findings highlight the promising potential of these botanical ratios in therapeutic applications, offering effective and safe options for managing oxidative stress and inflammation.

## Figures and Tables

**Table 1:** Characterizations of percent yield and phenolic and flavonoid compound contents in the Thai herbal recipe containing *Garcinia mangostana* Linn., *Curcuma comosa* Roxb., and *Acanthus ebracteatus* Vahl. with water and ethanol extraction following the Box‒Behnken design.

Run	*GM* (g)	*CC* (g)	*AE* (g)	Ethanol yield (%)	Water yield (%)	Phenolic of ethanol extraction (mg GAE/g)	Phenolic of water extraction (mg GAE/g)	Flavonoid of ethanol extraction (mg QE/g)	Flavonoid of water extraction (mg QE/g)
1	10	5	15	7.65	6.78	173.17	30.33	120.45	5.47
2	5	10	5	4.94	4.43	117.11	16.54	70.17	7.41
3	10	15	5	9.17	5.61	228.50	33.33	125.25	5.63
4	10	5	5	5.93	3.85	120.20	26.51	84.72	9.03
5	10	10	10	4.88	9.67	175.83	37.83	106.26	11.06
6	10	10	10	4.67	9.27	173.83	37.83	109.57	11.46
7	5	10	15	7.35	8.35	90.83	23.17	65.05	6.19
8	15	10	15	9.15	7.79	221.37	37.39	136.18	10.77
9	5	15	10	8.78	6.52	151.17	17.50	83.63	3.29
10	15	10	5	8.31	8.32	151.83	29.00	113.33	2.91
11	15	5	10	9.03	6.37	231.67	36.50	143.60	4.82
12	10	15	15	8.66	8.38	218.27	33.42	131.63	10.15
13	5	5	10	5.7	3.94	101.16	16.88	71.46	6.73
14	15	15	10	8.86	8.29	231.31	37.05	134.90	10.45
15	10	10	10	5.02	10.07	161.83	37.17	110.45	11.19

**Table 2:** Antioxidant inhibition and fractional percentage inhibitory index of the herbal combination at 250 *µ*g/mL.

Herb weights			Concentration (250 *µ*g/mL)			
*Garcinia mangostana* (g)	*Curcuma comosa* Roxb. (g)	*Acanthus ebracteatus* (g)	% DPPH radical scavenging	FPI	% ABTS	FPI
5	10	15	77.20 ± 1.42	0.4	83.70 ± 2.73	0.4
5	15	10	76.80 ± 2.22	0.4	90.511 ± 0.61	0.4
10	5	15	74.91 ± 1.65	0.4	91.25 ± 0.94	0.4
10	15	5	80.33 ± 1.29	0.5	92.20 ± 0.35	0.5
10	10	10	76.23 ± 1.14	0.4	91.89 ± 0.48	0.4
15	5	10	75.62 ± 1.34	0.4	91.04 ± 0.99	0.4
15	10	5	77.20 ± 1.10	0.4	88.96 ± 1.29	0.4
30			69.68 ± 2.48		75.11 ± 0.82	
	30		74.35 ± 1.78		79.57 ± 0.63	
		30	30.74 ± 1.03		50.11 ± 1.20	

**Table 3:** Anti-inflammatory properties of Thai herbal recipes containing *Garcinia mangostana* Linn., *Curcuma comosa* Roxb., and *Acanthus ebracteatus* Vahl. with water and ethanol extraction by controlling the total weight to 30 grams through an NO scavenger.

Plants			Percentage of anti-inflammation at 100 *µ*g/mL	
*GM* (g)	*CC* (g)	*AE* (g)	Water extraction	Ethanol extraction
5	10	15	53.47 ± 8.14	98.86 ± 1.12
5	15	10	26.30 ± 6.36	96.57 ± 1.62
10	5	15	52.31 ± 4.75	75.14 ± 1.71
10	15	5	26.88 ± 8.55	94.29 ± 1.44
10	10	10	64.74 ± 4.86	98.29 ± 0.93
15	5	10	63.01 ± 6.36	93.43 ± 1.71
15	10	5	54.62 ± 7.46	97.14 ± 1.95
30			15.90 ± 5.20	95.43 ± 2.16
	30		4.62 ± 2.75	96.86 ± 0.57
		30	17.44 ± 3.34	76.00 ± 1.44

**Table 4:** IC_50_ values and fractional percentage inhibitory indices of the anti-inflammatory properties of Thai herbal recipes containing *Garcinia mangostana* Linn., *Curcuma comosa* Roxb., and *Acanthus ebracteatus* Vahl. with water and ethanol extraction by controlling the total weight of the mixture to 30 grams through an NO scavenger.

*GM* (g)	*CC* (g)	*AE* (g)	Anti-inflammation (% inhibition)			IC_50_ (*µ*g/mL)
			10 *µ*g/mL	20 *µ*g/mL	50 *µ*g/mL	
5	10	15	44.84 ± 2.41	66.10 ± 1.56	98.86 ± 1.12	12.05 ± 0.94
5	15	10	33.73 ± 5.66	56.64 ± 4.69	96.57 ± 1.62	16.76 ± 2.35
10	5	15	33.99 ± 1.95	48.23 ± 4.29	75.14 ± 1.71	21.85 ± 3.85
10	15	5	33.20 ± 3.55	63.56 ± 4.29	94.29 ± 1.44	15.15 ± 1.37
10	10	10	45.50 ± 9.77	64.97 ± 4.15	98.29 ± 0.93	11.47 ± 4.08
15	5	10	34.79 ± 3.25	62.43 ± 4.45	93.43 ± 1.71	15.12 ± 1.50
15	10	5	24.74 ± 0.51	60.73 ± 3.61	97.14 ± 1.95	16.89 ± 0.76
30			35.32 ± 2.14	78.81 ± 3.24	95.43 ± 2.16	11.63 ± 1.30
	30		45.50 ± 3.19	71.89 ± 2.14	96.86 ± 0.57	13.51 ± 3.56
		30	29.10 ± 3.68	32.63 ± 4.50	76.00 ± 1.44	51.00 ± 5.41

## Data Availability

The data that support the findings of this study are available in the Supplementary Materials of this article.

## Supplementary Materials

Supplementary Materials Supplementary Table S1: LC-MS/MS analytical parameters of the Thai herbal combination of *GM-CC-AE* of 10-15-5; Supplementary Table S2: LC-MS/MS analytical parameters of the Thai herbal combination of *GM-CC-AE* of 5-10-15; Supplementary Table S3: the list of genes associated with the Thai herbal combination of *GM-CC-AE* (10-15-5), inflammation, and oxidative stress; Supplementary Table S4: target proteins of the top 7 compounds from the *GM-CC-AE* combination of 10-15-5; Supplementary Table S5: overlap between the top 7 compounds in the *GM-CC-AE* combination of 10-15-5 and target proteins of inflammation and oxidative stress; Supplementary Table S6: the list of genes associated with the Thai herbal combination of *GM-CC-AE* of 5-10-15, inflammation, and oxidative stress; Supplementary Table S7: overlap between the top 23 compounds in the *GM-CC-AE* combination (5-10-15) and target proteins of inflammation and oxidative stress.
