# Supplementary material for: Exploring Synergistic Inhibition of Inflammatory and Antioxidant Potential: Integrated In Silico and In Vitro Analyses of Garcinia mangostana, Curcuma comosa, and Acanthus ebracteatus
Source: Adv Pharmacol Pharm Sci. 2024 Sep 18;2024:8584015. doi: 10.1155/2024/8584015 (PMC11424873; doi:10.1155/2024/8584015)
Supplement: Supplementary Materials — Supplementary Table S1: LC-MS/MS analytical parameters of the Thai herbal combination of GM-CC-AE of 10-15-5; Supplementary Table S2: LC-MS/MS analytical parameters of the Thai herbal combination of GM-CC-AE of 5-10-15; Supplementary Table S3: the list of genes associated with the Thai herbal combination of GM-CC-AE (10-15-5), inflammation, and oxidative stress; Supplementary Table S4: target proteins of the top 7 compounds from the GM-CC-AE combination of 10-15-5; Supplementary Table S5: overlap between the top 7 compounds in the GM-CC-AE combination of 10-15-5 and target proteins of inflammation and oxidative stress; Supplementary Table S6: the list of genes associated with the Thai herbal combination of GM-CC-AE of 5-10-15, inflammation, and oxidative stress; Supplementary Table S7: overlap between the top 23 compounds in the GM-CC-AE combination (5-10-15) and target proteins of inflammation and oxidative stress. [file 8584015.f1.docx]

**Supplementary table S1** LC-MS/MS analytical parameter of Thai herbal combination of *GM-CC-AE* as 10:15:5

| **NO.** | **Identification** | **R_T_ (min)** | **[M-H] (m/z)** | **Score (DB)** | **Formula** | **Peak area (%)** |
| --- | --- | --- | --- | --- | --- | --- |
|  | **Phenolic** |  |  |  |  |  |
|  | Chlorogenic Acid | 5.732 | 353.0886 | 97.47 | C_16_ H_18_O_9_ | 1.1 |
|  | Esculetin | 6.433 | 177.0194 | 99.89 | C_9_ H_6_O_4_ | 0.03 |
|  | Dihydroxyphenylacetic acid | 6.734 | 167.0355 | 98.68 | C_8_ H_8_ O_4_ | 0.04 |
|  | 5,7-Dihydroxy-4-Methylcoumarin | 13.689 | 191.0354 | 97.75 | C_10_ H_8_O_4_ | 0.19 |
|  | dimethoxy Curcumin | 23.764 | 395.1512 | 95.53 | C_23_ H_24_ O_6_ | 24.44 |
|  | 8-Hydroxycarapinic Acid | 25.854 | 469.1881 | 94.01 | C_26_ H_30_ O_8_ | 8.59 |
|  | **Flavonoids** |  |  |  |  |  |
|  | Rutin | 8.893 | 609.1461 | 96.53 | C_27_ H_30_ O_16_ | 0.02 |
|  | Isovitexin | 9.367 | 431.099 | 90.94 | C_21_H_20_ O_10_ | 0.84 |
|  | Quercetin 3-galactoside | 9.571 | 463.0885 | 98.53 | C_21_H_20_O_12_ | 0.37 |
|  | Baicalin | 11.253 | 445.078 | 98.06 | C_21_ H_18_ O_11_ | 0.24 |
|  | Luteolin | 15.119 | 285.0418 | 93.31 | C_15_ H_10_ O_6_ | 0.64 |
|  | Quercetin | 15.309 | 301.0369 | 92.66 | C_15_ H_10_ O_7_ | 0.1 |
|  | Apigenin | 17.619 | 269.0465 | 95.33 | C_15_ H_10_ O_5_ | 0.23 |
|  | **Terpenes/ Terpenoids** |  |  |  |  |  |
|  | Diacetyldideisovaleryl-Rhodomyrtoxin | 25.314 | 343.0837 | 93.94 | C_18_ H_16_ O_7_ | 0.33 |
|  | Rhodomyrtoxin | 36.133 | 427.1782 | 90.76 | C_24_ H_28_ O_7_ | 0.78 |
|  | α-Mangostin | 38.833 | 409.167 | 94.52 | C_24_ H_26_ O_6_ | 14.14 |
|  | **Lignans** |  |  |  |  |  |
|  | 4'-Demethylepipodophyllotoxin | 31.767 | 399.1097 | 94.51 | C_21_ H_20_ O_8_ | 0.2 |
|  | **Steroids** |  |  |  |  |  |
|  | 4-Androsten-3,17-dione 19-aldehyde | 20.355 | 299.1663 | 95.53 | C_19_ H_24_ O_3_ | 0.37 |
|  | **Fatty acids** |  |  |  |  |  |
|  | Pinolenic Acid | 40.191 | 277.2184 | 93.15 | C_18_ H_30_ O_2_ | 1.1 |
|  | (+)-Isomyristic acid | 40.555 | 227.2022 | 96.13 | C_14_ H_28_ O_2_ | 1.79 |
|  | Stearic acid | 40.681 | 283.2649 | 96.41 | C_18_ H_36_ O_2_ | 0.18 |
|  | Pentadecylic acid | 42.087 | 241.218 | 95.29 | C_15_ H_30_ O_2_ | 2.48 |
|  | 10E,12Z-Octadecadienoic acid | 42.413 | 279.2339 | 94.52 | C_18_ H_32_ O_2_ | 0.53 |
|  | **other** |  |  |  |  |  |
|  | Sulfamethoxypyridazine | 9.772 | 279.0551 | 91.77 | C_11_H_12_N_4_O_3_S | 0.82 |
|  | D-Mannonate | 1.938 | 195.0518 | 96.83 | C_6_ H_12_ O_7_ | 0.42 |
|  | Quinic acid | 1.951 | 191.057 | 95.59 | C_7_ H_12_ O_6_ | 0.01 |

**Supplementary table S2** LC-MS/MS analytical parameter of Thai herbal combination of *GM-CC-AE* as 5:10:15

| **NO.** | **Identification** | **R_T_ (min)** | **[M-H] (m/z)** | **Score (DB)** | **Formula** | **Peak area (%)** |
| --- | --- | --- | --- | --- | --- | --- |
|  | **Phenolic** |  |  |  |  |  |
| 1. | Galactonic acid | 1.674 | 195.0519 | 95.92 | C_6_ H_12_ O_7_ | 0.02 |
| 2. | 3'-Glucosyl-2',4',6'-trihydroxyacetophenone | 5.709 | 329.0889 | 95.48 | C_14_ H_18_O_9_ | 0.20 |
| 3. | Chlorogenic Acid | 5.163 | 353.0886 | 96.93 | C_16_ H_18_O_9_ | 0.07 |
| 4. | Hydrojuglone glucoside | 6.783 | 337.0943 | 93.76 | C_16_ H_18_ O_8_ | 0.03 |
| 5. | 5Z-Caffeoylquinic acid | 6.971 | 353.0884 | 98.14 | C_16_ H_18_ O_9_ | 0.03 |
| 6. | 3-O-Caffeoyl-4-O-methylquinic acid | 7.549 | 367.104 | 97.24 | C_17_ H_20_ O_9_ | 0.01 |
| 7. | 2-Hydroxy-6-oxo-6-(2-hydroxyphenoxy)-hexa-2,4-dienoate | 7.611 | 309.0623 | 97.06 | C_12_ H_10_ O_6_ | 0.02 |
| 8. | 1,3,6-Trihydroxy-5-methoxyxanthone | 9.363 | 273.0412 | 89.09 | C_14_ H_10_ O_6_ | 0.05 |
| 9. | Liqcoumarin | 11.817 | 263.0568 | 96.69 | C_12_ H_10_ O_4_ | 0.09 |
| 10. | 3-Methoxy-4-hydroxyphenylethylene glycol | 12.47 | 183.0664 | 99.66 | C_9_ H_12_ O_4_ | 0.18 |
| 11. | 1-(4-Hydroxy-3,5-dimethoxyphenyl)-2-[2-methoxy-4-(1-propenyl)phenoxy]-1-propanol | 17.329 | 373.1672 | 92.1 | C_21_ H_26_ O_6_ | 0.11 |
| 12. | Tetrahydrogambogic Acid | 18.044 | 631.3288 | 97.29 | C_38_ H_48_ O_8_ | 0.42 |
| 13. | 4-(3-Hydroxy-7-phenyl-6-heptenyl)-1,2-benzenediol | 19.664 | 297.1509 | 94.35 | C_19_ H_22_ O_3_ | 0.19 |
| 14. | Pseudolaric Acid B | 20.404 | 431.1731 | 91.53 | C_23_ H_28_ O_8_ | 1.36 |
| 15. | Broussinol | 22.337 | 325.1454 | 92.46 | C_20_ H_22_ O_4_ | 0.34 |
| 16. | 5,5-Diisopropyl-2,2'-dimethylbiphenyl-3,3',4,4'-tetrone | 22.991 | 325.1462 | 92.45 | C_20_ H_22_ O_4_ | 0.05 |
| 17. | dimethoxy Curcumin | 23.771 | 395.1509 | 94.71 | C_23_ H_24_ O_6_ | 4.54 |
| 18. | 8-Hydroxycarapinic Acid | 25.866 | 469.188 | 91.75 | C_26_ H_30_ O_8_ | 1.02 |
| 19. | 7-Deoxyadriamycinone | 26.205 | 397.0943 | 94.63 | C_21_ H_18_ O_8_ | 0.25 |
| 20. | Mangostinone | 26.355 | 379.1563 | 92.41 | C_23_ H_24_ O_5_ | 1.07 |
| 21. | Lobaric Acid | 28.866 | 455.1722 | 94.47 | C_25_ H_28_ O_8_ | 0.65 |
| 22. | 7-(4-Hydroxyphenyl)-1-phenyl-4-hepten-3-one | 29.067 | 279.1403 | 94.85 | C_19_ H_20_ O_2_ | 0.87 |
| 23. | Garcinone C | 29.343 | 413.1617 | 91.53 | C_23_ H_26_ O_7_ | 2.3 |
| 24. | Avobenzone | 30.372 | 309.1501 | 98.51 | C_20_ H_22_ O_3_ | 2.07 |
| 25. | Rhodomyrtoxin | 35.859 | 427.1778 | 93.34 | C_24_ H_28_ O_7_ | 0.88 |
| 26. | (E)-4-(3,7-Dimethyl-2,6-octadienyl)-1,3,5-trihydroxyxanthone | 36.499 | 379.1561 | 94.9 | C_23_ H_24_ O_5_ | 0.77 |
| 27. | Gerberinol | 37.503 | 363.0882 | 93.43 | C_21_ H_16_ O_6_ | 0.1 |
| 28. | Mangostenol | 38.847 | 425.1626 | 91.16 | C_24_ H_26_ O_7_ | 1.02 |
| 29. | Garcinone E | 39.813 | 523.2336 | 98.12 | C_28_ H_32_ O_6_ | 0.22 |
| 30. | Mangostenone B | 42.299 | 461.1979 | 90.84 | C_28_ H_30_ O_6_ | 0.1 |
|  | **Flavonoids** |  |  |  |  |  |
| 31. | Catechin 3',7-diglucoside | 5.816 | 659.1842 | 97 | C_27_ H_34_ O_16_ | 0.31 |
| 32. | Mangiferin | 6.143 | 421.0782 | 96.28 | C_19_ H_18_ O_11_ | 0.05 |
| 33. | Vitexin 4'-O-galactoside | 6.306 | 593.1518 | 96.72 | C_27_ H_30_ O_15_ | 0.02 |
| 34. | APIIN | 7.448 | 563.1412 | 98.6 | C_26_ H_28_ O_14_ | 0.02 |
| 35. | Kaempferol 4'-glucoside | 7.674 | 447.0939 | 95.56 | C_21_ H_20_ O_11_ | 0.04 |
| 36. | Cichoriin | 8.076 | 339.0732 | 95.28 | C_15_ H_16_ O_9_ | 0.03 |
| 37. | 4'-Hydroxy-5,7,2'-trimethoxyflavanone 4'-rhamnosyl-(1->6)-glucoside | 11.089 | 637.2139 | 98.38 | C_30_ H_38_ O_15_ | 0.09 |
| 38. | Kiwiionoside | 11.792 | 451.2194 | 92.91 | C_19_ H_34_ O_9_ | 0.1 |
| 39. | 5-Hydroxy-7,8-dimethoxyflavanone 5-rhamnoside | 12.345 | 445.1515 | 95.44 | C_23_ H_26_ O_9_ | 0.04 |
| 40. | 3beta,4beta,5-Trimethoxy-4'-hydroxy- (6:7)-2,2-dimethylpyranoflavan | 13.512 | 443.1724 | 95.4 | C_23_ H_26_ O_6_ | 0.09 |
| 41. | 2',4',4-Trihydroxy-3'-prenylchalcone 4'-O-glucoside | 14.403 | 485.182 | 98.42 | C_26_ H_30_ O_9_ | 0.05 |
| 42. | Apigenin | 17.567 | 269.0467 | 90.77 | C_15_ H_10_ O_5_ | 0.56 |
| 43. | Diosmetin | 18.22 | 299.0573 | 92.06 | C_16_ H_12_ O_6_ | 0.23 |
| 44. | 2,8-Dihydroxy-3,9,10-trimethoxypterocarpan | 18.321 | 345.0991 | 94.77 | C_18_ H_18_ O_7_ | 0.37 |
| 45. | 2',4'-Dihydroxy-7-methoxy-8-prenylflavan | 20.429 | 339.1613 | 96 | C_21_ H_24_ O_4_ | 0.27 |
| 46. | (+)-Tephropurpurin | 42.324 | 423.147 | 90.86 | C_24_ H_24_ O_7_ | 0.79 |
| 47. | 5-Hydroxy-4,6,4'-trimethoxyaurone | 22.036 | 327.0888 | 92.01 | C_18_ H_16_ O_6_ | 0.44 |
| 48. | 8-Prenylafzelechin 5-methyl ether | 23.242 | 355.1564 | 92.61 | C_21_ H_24_ O_5_ | 0.26 |
| 49. | Sphenostylin A | 24.886 | 443.173 | 91.76 | C_23_ H_26_ O_6_ | 0.05 |
| 50. | Derrubone | 25.351 | 365.1043 | 92.17 | C_21_ H_18_ O_6_ | 0.11 |
| 51. | 5,7,4'-Trimethoxyflavone | 27.899 | 311.0936 | 95.7 | C_18_ H_16_ O_5_ | 0.31 |
| 52. | 7-Hydroxy-5,4'-dimethoxy-8-methylisoflavone 7-O-rhamnoside | 27.058 | 457.1513 | 93.68 | C_24_ H_26_ O_9_ | 0.34 |
| 53. | Quercetin Tetramethyl (5,7,3',4') Ether | 28.414 | 357.0989 | 93.16 | C_19_ H_18_ O_7_ | 0.39 |
| 54. | Semilicoisoflavone B | 31.879 | 351.0887 | 91.4 | C_20_ H_16_ O_6_ | 0.2 |
| 55. | 5-Hydroxy-7,2',5'-trimethoxyflavone | 31.979 | 327.0884 | 94.09 | C_18_ H_16_ O_6_ | 0.08 |
| 56. | Ovalifolin | 32.231 | 405.1356 | 94.73 | C_22_ H_18_ O_4_ | 0.12 |
| 57. | Laxiflorin | 34.102 | 413.1618 | 95.28 | C_23_ H_26_ O_7_ | 1.59 |
| 58. | 3-(2,4-Dihydroxyphenyl)-8,9-dihydro-5-hydroxy-8-(1-methylethenyl)-4H-furo[2,3-h]-1-benzopyran-5-one | 34.227 | 351.089 | 91.51 | C_20_ H_16_ O_6_ | 0.86 |
| 59. | Derrusnin | 34.265 | 355.0835 | 94.92 | C_19_ H_16_ O_7_ | 0.84 |
| 60. | Lonchocarpol B | 34.992 | 441.1932 | 92.82 | C_25_ H_30_ O_7_ | 0.23 |
| 61. | 2-Methyl-5,7,8-Trimethoxyisoflavone | 35.495 | 325.1093 | 92.77 | C_19_ H_18_ O_5_ | 0.57 |
| 62. | Angustone C | 36.144 | 419.1511 | 92.5 | C_25_ H_24_ O_6_ | 1.84 |
| 63. | Gedunin | 37.265 | 481.2253 | 91.28 | C_28_ H_34_ O_7_ | 0.68 |
| 64. | Heteroartonin A | 42.199 | 451.1781 | 92.32 | C_26_ H_28_ O_7_ | 0.88 |
|  | **Alkaloids** |  |  |  |  |  |
| 65. | Pteridine | 1.962 | 191.0571 | 93.95 | C_6_ H_4_ N_4_ | 0.01 |
| 66. | Blepharin | 5.365 | 326.0893 | 93.76 | C_14_ H_17_NO_8_ | 0.05 |
| 67. | Sulfamethoxypyridazine | 9.683 | 279.0558 | 93.84 | C_11_H_12_N_4_ O_3_S | 0.1 |
| 68. | Amataine | 23.568 | 715.35 | 96.99 | C_43_ H_48_ N_4_ O_6_ | 0.14 |
| 69. | Ro 31-7549 | 28.665 | 443.1726 | 92.3 | C_24_ H_22_ N_4_ O_2_ | 2.82 |
|  | **Glucosides** |  |  |  |  |  |
| 70. | 1-O-E-Cinnamoyl-(6-arabinosylglucose) | 2.226 | 487.1464 | 95.58 | C_20_ H_26_ O_11_ | 0.04 |
| 71. | Verbasoside | 3.582 | 461.1671 | 95.68 | C_20_ H_30_ O_12_ | 0.02 |
| 72. | 1-Octen-3-yl primeveroside | 5.565 | 467.2138 | 96.08 | C_19_ H_34_ O_10_ | 0.03 |
| 73. | Benzyl O-[arabinofuranosyl-(1->6)-glucoside] | 6.205 | 401.1459 | 97.2 | C_18_ H_26_ O_10_ | 0.02 |
| 74. | Plantamajoside | 7.034 | 639.1947 | 95.74 | C_29_ H_36_ O_16_ | 0.07 |
| 75. | Cincassiol B | 7.373 | 445.2081 | 91.24 | C_20_ H_32_ O_8_ | 0.01 |
| 76. | Cladrastin 7-O-laminaribioside | 9.256 | 651.1932 | 94.43 | C_30_ H_36_ O_16_ | 0.41 |
| 77. | Isoacteoside | 9.432 | 623.2003 | 93.97 | C_29_ H_36_ O_15_ | 0.5 |
| 78. | Macrocarposide | 9.897 | 449.1104 | 95.27 | C_21_ H_22_ O_11_ | 0.32 |
| 79. | Magnoloside A | 10.248 | 623.1994 | 95.66 | C_29_ H_36_ O_15_ | 0.04 |
| 80. | Kanzonol M | 10.486 | 443.172 | 95.92 | C_23_ H_26_ O_6_ | 0.05 |
| 81. | Miconioside A | 10.763 | 667.2244 | 95.83 | C_29_ H_36_ O_14_ | 0.03 |
| 82. | Multigilin | 14.102 | 359.1508 | 97.97 | C_20_ H_24_ O_6_ | 0.09 |
|  | **Terpenes/Terpenoids** |  |  |  |  |  |
| 83. | Artemisinin | 7.122 | 281.1397 | 98.23 | C_15_ H_22_ O_5_ | 0.06 |
| 84. | 3-Epigibberellin A1 | 10.21 | 347.1517 | 92.15 | C_19_ H_24_ O_6_ | 0.08 |
| 85. | Gibberellin A70 | 12.094 | 331.1567 | 92.93 | C_19_ H_24_ O_5_ | 0.14 |
| 86. | Neobanone | 13.349 | 381.099 | 95.38 | C_21_ H_18_ O_7_ | 0.06 |
| 87. | Gibberellin A51-catabolite | 13.437 | 329.1405 | 91.54 | C_19_ H_22_ O_5_ | 0.1 |
| 88. | Urolithin D | 13.763 | 259.0258 | 95.23 | C_13_ H_8_ O_6_ | 0.04 |
| 89. | 6-Acetylpicropolin | 14.429 | 459.1669 | 97.64 | C_24_ H_28_ O_9_ | 0.03 |
| 90. | Baccatin III | 14.579 | 585.234 | 97.41 | C_31_ H_38_ O_11_ | 0.09 |
| 91. | Erioflorin acetate | 15.119 | 389.1618 | 93.28 | C_21_ H_26_ O_7_ | 0.23 |
| 92. | Gibberellin A20 | 15.722 | 331.1561 | 96.89 | C_19_ H_24_ O_5_ | 0.46 |
| 93. | Urolithin C | 16.927 | 243.0307 | 96.66 | C_13_ H_8_ O_5_ | 0.43 |
| 94. | Gambogic acid | 17.215 | 627.2973 | 95.78 | C_38_ H_44_ O_8_ | 0.42 |
| 95. | 2,3-Didehydrogibberellin A9 | 17.404 | 313.1456 | 96.65 | C_19_ H_22_ O_4_ | 0.42 |
| 96. | Gibberellin A9 | 18.534 | 315.1613 | 95.68 | C_19_ H_24_ O_4_ | 0.3 |
| 97. | 10-Hydroxymelleolide | 21.296 | 415.1781 | 90.91 | C_23_ H_28_ O_7_ | 0.53 |
| 98. | Gibberellin A9 | 23.643 | 315.1609 | 97.53 | C_19_ H_24_ O_4_ | 0.75 |
| 99. | Asarinin (-) | 26.732 | 353.1045 | 93.29 | C_20_ H_18_ O_6_ | 0.19 |
| 100. | Mundoserone | 29.444 | 341.1042 | 95.44 | C_19_ H_18_ O_6_ | 2.61 |
| 101. | Idarubicinol aglycone | 29.732 | 369.0996 | 91.66 | C_20_ H_18_ O_7_ | 1.57 |
| 102. | Oxyisocyclointegrin | 30.071 | 383.1151 | 94.36 | C_21_ H_20_ O_7_ | 0.99 |
| 103. | Normammein | 30.184 | 357.172 | 94.36 | C_21_ H_26_ O_5_ | 0.28 |
| 104. | 6-Prenylisocaviunin | 33.21 | 441.1568 | 95.57 | C_24_ H_26_ O_8_ | 3.54 |
| 105. | 2-Prenyl-6a-hydroxyphaseollidin | 35.294 | 407.1877 | 93.87 | C_25_ H_28_ O_5_ | 0.47 |
| 106. | Anthothecol | 35.57 | 479.2095 | 92.94 | C_28_ H_32_ O_7_ | 0.21 |
| 107. | Austinol | 35.671 | 457.1879 | 93.61 | C_25_ H_30_ O_8_ | 0.52 |
| 108. | Exiguaflavanone M | 37.34 | 441.193 | 95.29 | C_25_ H_30_ O_7_ | 0.07 |
| 109. | Lepidissipyrone | 37.516 | 421.1302 | 93.34 | C_24_ H_22_ O_7_ | 1.1 |
| 110. | Dihydroamorphigenin | 38.357 | 411.1461 | 93.75 | C_23_ H_24_ O_7_ | 2.11 |
| 111. | α-Mangostin | 39.173 | 409.1669 | 95.43 | C_24_ H_26_ O_6_ | 0.61 |
| 112. | 6,11-Dihydroxy-3-methyl-3-(4-methyl-3-pentenyl)-3H,7H-pyrano[2,3-c]xanthen-7-one | 40.366 | 377.1406 | 91.34 | C_23_ H_22_ O_5_ | 0.02 |
| 113. | Pomiferin | 41.42 | 419.1512 | 92.83 | C_25_ H_24_ O_6_ | 0.59 |
|  | **Steroids** |  |  |  |  |  |
| 114. | Estrone glucuronide | 22.124 | 445.1883 | 93.63 | C_24_ H_30_ O_8_ | 0.35 |
| 115. | Prednisone | 24.158 | 357.172 | 90.32 | C_21_ H_26_ O_5_ | 0.1 |
| 116. | Quassin | 24.397 | 387.1824 | 93.07 | C_22_ H_28_ O_6_ | 0.11 |
| 117. | 4-Androsten-3,17-dione 19-aldehyde | 24.598 | 299.1663 | 95.54 | C_19_ H_24_ O_3_ | 0.26 |
| 118. | 17β-hydroxy Wortmannin | 30.209 | 429.157 | 94.47 | C_23_ H_26_ O_8_ | 1.42 |
| 119. | 13-Ethyl-6a,17-dihydroxy-18,19-dinor-17a-pregna-4,9,11-trien-20-yn-3-one | 31.528 | 323.1668 | 93.62 | C_21_ H_24_ O_3_ | 0.11 |
| 120. | Descinolone acetonide | 39.977 | 463.2149 | 95.34 | C_24_ H_31_ FO_5_ | 0.35 |
| 121. | 9alpha-Fluoro-6alpha-methylprednisolone 21-acetate | 39.989 | 479.2078 | 97.37 | C_24_ H_31_ FO_6_ | 0.37 |
| 122. | 11(R)-HEDE | 41.722 | 323.2599 | 96.76 | C_20_ H_36_ O_3_ | 0.16 |
|  | **Lignans** |  |  |  |  |  |
| 123. | (+)-Lyoniresinol 9-glucoside | 8.051 | 581.2246 | 96.87 | C_28_ H_38_ O_13_ | 0.01 |
| 124. | Duartin, Dimethyl Ether | 14.905 | 359.1507 | 97.08 | C_20_ H_24_ O_6_ | 0.21 |
| 125. | 4'-Demethylepipodophyllotoxin | 24.748 | 399.1098 | 94.03 | C_21_ H_20_ O_8_ | 1.03 |
| 126. | 8-Acetoxy-4'-methoxypinoresinol | 27.134 | 429.1576 | 90.69 | C_23_ H_26_ O_8_ | 0.18 |
| 127. | Deoxygomisin A | 27.874 | 399.1827 | 92.03 | C_23_ H_28_ O_6_ | 1.1 |
|  | **Fatty acid** |  |  |  |  |  |
| 128. | 11,12,13-trihydroxy-9-octadecenoic acid | 19.048 | 329.2347 | 93.5 | C_18_ H_34_ O_5_ | 0.22 |
| 129. | 3,12-dihydroxy palmitic acid | 20.003 | 287.2235 | 94.41 | C_16_ H_32_ O_4_ | 0.21 |
| 130. | 13-OxoODE | 34.591 | 293.2131 | 96.38 | C_18_ H_30_ O_3_ | 0.38 |
| 131. | 16-hydroxy hexadecanoic acid | 40.115 | 271.2288 | 95.1 | C_16_ H_32_ O_3_ | 0.62 |
| 132. | Myristic acid | 40.567 | 227.2027 | 94.24 | C_14_ H_28_ O_2_ | 1.14 |
| 133. | Stearic acid | 41.006 | 283.2651 | 96 | C_18_ H_36_ O_2_ | 0.13 |
| 134. | Pentadecylic acid | 42.601 | 241.2182 | 93.87 | C_15_ H_30_ O_2_ | 0.13 |
|  | **Other** |  |  |  |  |  |
| 135. | 2-Benzoxazolol | 7.272 | 134.0247 | 98.22 | C_7_ H_5_ NO_2_ | 0.02 |
| 136. | Corchoionoside B | 7.411 | 459.1874 | 98.58 | C_19_ H_28_ O_9_ | 0.01 |
| 137. | Cyclic de-hypoxanthine futalosine | 9.758 | 293.0677 | 94.74 | C_14_ H_14_ O_7_ | 0.04 |
| 138. | 4'-Hydroxyfenoprofen glucuronide | 11.441 | 433.1153 | 93.56 | C_21_ H_22_ O_10_ | 0.19 |
| 139. | Lepidine E | 11.579 | 345.1354 | 96.26 | C_20_ H_18_ N_4_ O_2_ | 0.03 |
| 140. | Idebenone | 11.993 | 267.088 | 93.84 | C_13_ H_16_ O_6_ | 0.04 |
| 141. | 17a-Ethynylestradiol 16-glucuronide | 19.827 | 487.1986 | 94.12 | C_26_ H_32_ O_9_ | 0.27 |
| 142. | 16-Hydroxy-4-oxoretinoic acid | 20.806 | 329.1766 | 95.79 | C_20_ H_26_ O_4_ | 0.51 |
| 143. | Gancaonin D | 28.715 | 383.1152 | 93.88 | C_21_ H_20_ O_7_ | 0.27 |
| 144. | Acitretin Ro 23-4750 | 31.063 | 341.1771 | 92.93 | C_21_ H_26_ O_4_ | 1.76 |
| 145. | Sudan III | 32.946 | 411.1473 | 92.55 | C_22_ H_16_ N_4_ O | 0.74 |
| 146. | Scarlet Red | 33.436 | 425.163 | 96.82 | C_24_ H_20_ N_4_ O | 0.9 |
| 147. | Archangelicin | 33.687 | 425.1618 | 95.34 | C_24_ H_26_ O_7_ | 0.97 |
| 148. | Disenecionyl cis-khellactone | 34.779 | 425.1619 | 93.89 | C_24_ H_26_ O_7_ | 0.76 |
| 149. | Devazepide | 38.282 | 407.1516 | 94.37 | C_25_ H_20_ N_4_ O_2_ | 0.63 |
| 150. | 9-(3,4-Dimethoxyphenyl)-2-methoxy-1H-phenalen-1-one | 41.835 | 405.1353 | 95.94 | C_22_ H_18_ O_4_ | 0.28 |

**Supplementary table S3** The list of genes of Thai herbal combination of *GM-CC-AE* as 10:15:5, inflammation and oxidative stress.

| **NO.** | **Names** | **Total** | **Gene targets** |
| --- | --- | --- | --- |
| 1 | (10-15-5)-Inflammation-oxidant | 20 | XDH ALOX5 ELANE NFE2L2 TNF MPO CASP1 TRPA1 PTGS2 OLR1 IL6 MAPK1 FOS MAPK8 ICAM1 MAPK3 SIRT1 PTGS1 TLR2 JUN |
| 2 | (10-15-5)-Oxidant | 25 | PRKCG NOS2 REST CTSV MAPT BLM CTNNB1 SNCA KEAP1 VCP ACHE CASP3 ACE GSR CACNA1C BACE1 FABP1 MDM2 SLC1A1 G6PD IGF1R ERO1A AKR1B1 RAD51 APP |
| 3 | (10-15-5)-Inflammation | 66 | MMP2 PLAU EP300 NLRP3 HSD11B2 CGAS NR1H3 IMPDH2 DPP4 CXCR2 GRIK2 CASP8 PPARG MAPK14 IKBKG CCR2 CREBBP GGPS1 ITGB6 SELP TLR8 ITGAV JAK2 CHUK TNFRSF1A STAT3 F2 CSNK2B STING1 ESR1 GSK3B MMP1 STAT1 MMP12 PTGER4 TRPV1 VDR NR3C2 ABCG2 NAMPT NOD1 PIK3CG EGFR IKBKB SYK F10 HPRT1 HIF1A RELA CFTR PTPN6 XBP1 DPEP1 TLR4 TLR9 JAK1 FABP4 AKT1 CCR5 MIF NFKB1 NOD2 P2RX7 LTA4H S1PR3 MMP9 |
| 4 | Inflammation-Oxidant | 31 | CERNA3 PINK1 MIR7-3HG BDNF-AS HMCN1 CFH CRP SELE MIR29B1 TRA-TGC7-1 H19 MIR141 TRPM2 VEGFA TIMP1 CCL2 PARP1 HMOX1 PRKN VNN1 CAV1 MIR34C HFE TP53 PKM SOD2-OT1 TMX2-CTNND1 LINC01672 ALB SCARNA5 ADIPOQ |
| 5 | (10-15-5) | 750 | PTGER3 ERBB4 ZAP70 PRCP BBOX1 CYP26A1 Mmp2 TNIK PDF HSD17B1 CLK4 PRE7 GSTA1 HSD17B10 NAALAD2 POLK PLA2G2C SLC6A11 NTSR1 CA13 MMP13 Dusp6 CACNA2D1 KDM4D Grm4 SLC6A4 POLI GNAI3 FGR ND1 FGFR1 CHRM5 GALK1 MET SERPINA6 MTNR1B PTK2B Ca13 agrA CASP6 GRM2 Prkch SLCO2B1Ãƒâ€šÃ‚Â  pepN fabG luxR ARG SLC22A2 FASN TRPM8 TAOK3 PTPN22 ITGA3 AKR1B10 CDC25B LPAR4 PTPN12 ACVRL1 S1PR2 PRKCB RXRA MAP3K5 GRM8 C5AR1 CYP2C19 SMO GRK6 CA14 BMPR1A ROCK2 ACACA TLR1 TEK UGT2B7 GRK5 ACAT1 NUDT1 PTPN11 LNPEP KDM5C PDCD4 PTGDR CES2 SELL PLCG1 PLK1 HTR2C GPR55 HTR7 BHMT AVPR1B PLA2G2A FOLH1 MAOA HDAC5 NR2E3 psbA PIN1 SLCO2A1 HSPD1 PRKCI CDK1 Abcc2 PTK2 CYSLTR2 pol ADH5 SHBG P2RX3 DNM1 FABP5 CHEK1 BCHE RASD2 KIF11 HCRTR2 MMP16 SLC40A1 DRD3 HAO1 POLM PDE6D PIK3CB PRE2 SLC22A6 ICL1 GCK DNMT3B THRB CELA1 EDNRA SYN1 KCNH2 SND1 F9 ESRRA LDHB CYP1A1 ATR TNNI3 CAPN1 Ces2c GR3 CDC25C KDM5A MAPK10 sssIM SCN4A KDM2A Ptgs2 KRAS PTGES ERAP1 ATG4B OPRK1 CHRM1 APLNR TBXA2R ALOX15 CACNA1B Slco2b1 GLA Lef1 RARB CALCA PRKCD NQO2 GAPG TMPRSS6 TNNC1 AKR1C2 EST1 RET GSTP1 TCF4 GABRQ GABRG1 AHCY AURKA ASAH1 ADRA1A PTGER1Ãƒâ€šÃ‚Â  PTPN2 CHRNB4 PARP14 KISS1R PTGFR PTGER1 SMPD2 CNR1 ABCB1 AHR DHFR LOX1.4 EcR NR3C1 ITGB7 Fasn KLKB1 GAA GNAO1 HDAC3 FAAH ECE1 AURKB PNLIP MTOR GABBR1 CHRM4 SCN3A CYP2A6 PDE9A Grm8 KDM1A SPPS BRD4 CYP19A1 SLC22A8 SLC22A1 AOC3 FABP3 Slc6a8 RORB PDGFRB ADRB1 Cdc25b GRM6 CYP4A4 ADH7 CMA1 CACNA1H HCRTR1 PLA2G4A fbpC SENP6 ITGB1 DRD2 AKR1C4 MAP2K2 ABCC2 TUBA1A PSMB2 KLK2 ADRA1B NEU3 aroB AMY2A cqsS FNTB GABRB2 LDHA EDNRB TACR2 DPP7 SCD EGLN1 Gabbr2 NR1H2 DGAT1 S1PR4 CPT1B ERVW-4 SPHK1 TNNT2 PLA2G4C PHGDH HCAR2 CA9 TBXAS1 GPR174 FDFT1 APOBEC3A PSENEN SQLE ADAM10 ADH1C P2RY12 CYP2C9 SLC6A13 CNOT7 HK1 UTS2R MCHR1 NCSTN GCGR LOX1.1 CCND1 CA5A PRKDC COL4A3BP KDM6B CAN2 ACLY BMP4 CDK4 AGTR1 MC1R GABRA2 CSNK2A2 GBA MELK GRM1 PDE4D ITK CASP7 PDE3A CCKBR ITGB2 KAT5 CTSD STAT6 ENPP2 PIK3CD MME APEX1 fabZ GSP PTPA PSEN2 ADRA1D NTRK3 PTGIR PLK4 RXFP1 lasR MMEL1 CHKA SOAT1 FFAR1 MT-ND1 Msr1 PLA2G10 dxs MTNR1A MCL1 NS AMPD3 ADCYAP1R1 CA12 ADA DOT1L SMYD2 TRPM5 POLB EPHX2 PTPN7 LPAR2 KARS CPT2 PTGDR2 SLC2A1 METAP1 NR2F2 LTB4R TDP1 HSP90AA1 TRIM24 ACAT CA7 FPR1 CCR4 PTPRC KDM4E ITGA4 HMGCR PPARD KDM4A NPY5R PRKCE SLC37A4 MST1R NEU2 CPA1 MGAM RXRG GGH PRKAA2 glmU KCNK2 KDR PAM PARP15 HPD GRM3 GLO1 MC4R FLT1 ERAP2 HTR1A ALOX12 RARA PTPRS MKNK2 PTPN1 MMP3 LPAR3 AXL PPARA CNR2 Slc6a13 F13A1 MGLL RORA ALOX15B DUSP3 KDM7A TTR MEP2 CCR1 HTR2B CXCR1 NUS1 PSMG3 MAOB ITGA2B PTGER2 CYP1A2 MMP8 Polb HDAC4 LYN GLRA1 RBP4 GSTM1 LGMN CA4 GPR183 HSD17B3 Lss PYGL CTSG CHRNA1 ACR Slc22a20 FPGS KLK7 ALOX5AP DNMT1 HTR1B TNK1 P2RY6 PLA2G1B FTO CYP27B1 ADRA2B SUV39H1 PLA2G5 MMP14 PLAT KCNK9 Sqle NPC1L1 GNAI1 POLL NQO1 FFAR4 CYP4F2 GSTK1 S1PR1 SIRT5 CYSLTR1 PIK3CA GPBAR1 ADORA1 NR4A1 DCUN1D1 KDM4C SLC6A5 ERBB2 CREB1 DDIT3 LGALS3 SRD5A1 GRIN1 NAAA KCNA3 AGPAT2 PPO2 FPPS APH1A SCN2A XPO1 MCOLN3 CPT1BÃƒâ€šÃ‚Â  GRM5 TTL ENPEP GPR34 NEU4 CCKAR LPAR5 TIMM23 OPRD1 HK2 Grm6 KLF5 EPHX1 Gsk3b lpxC PAOX PDE5A TOP2A HDAC8 KLK5 NRG3 GPR35 ACVR1 ABAT BCL2L1 ADORA3 ABL1 GZMB ADK CDC45 SLC9A1 PDE7A HDAC2 FabI Slc6a11 CA2 hdaH HSP90AB1 PAFAH1B2 SLC25A20 HSP90B1 Naaa DPP9 GUSB HNF4A CHEK2 RNPEP FBP1 DRD4 CTDSP1 CDK5R1 TERT GABRR1 PAX8 NTSR2 Akr1b1 MC5R ANPEP OXER1 ADH1A P2RY10 HDAC11 INMT HSF1 GRIK3 PDE10A METAP2 TREH KMT2A ST14 SRC blaIMP-1 NR0B2 CDK2 PDE4B PGR CHRM2 GPR84 NR1H4 ODC1 resT PHF8 TFPI HTR1D THRA POLH Folh1 PTGES2 OPRM1 ACER2 DPP8 CA3 CYP26B1 TSPAN1 GFER GABRB3 lipN PLG TYR nanH SERPINE1 SLC13A5 ARG2 WDR5 Abcb1a RARG GLS STS ACVR1B SLC1A3 PTAFR NOX1 LPAR6 OR51E2 traR INSR TGFBR2 Prep PDK1 YARS MARK4 FNTA ACP1 CASP9 AKR1C1 ADH1B DAGLA DYRK1A CDK5 CA5B Gpr34 OGA Hsd17b3 CPT1 JAK3 APH1B GABRG2 GCG QRFPR F5 S1PR5 gag-pol ENPP1 P2RX4 PRKCH PIK3R1 ACACB PDGFRA MPEG1 APOBEC3G CISD1 SLC5A1 FPR2 EIF4H LAP3 GNPAT IMPDH1 PRKACA SLC22A12 RORC NMT1 IDH1 CAMKK2 PRKAB1 HDAC10 HDAC1 HK KLK1 PLB1 SLC5A2 ULK3 MAP2K1 luxP ABCC1 phzR ITGB3 BMPR1B CHRM3 GABBR2 ASF1A CTSA HPSE SAE1 Jhe HSD11B1 AKR1C3 PSMB1 IGFBP3 GAK CPT1A PTK6 murF DNTT F3 FUT7 NR1I2 LMNA AMPD2 PYGM FPRL2 plc PDE1B AKR1A1 PSEN1 ENGASE PLA2G4B LPAR1 CASP2 CA1 Gabrg1 ERN1 CSF1R ADAMTS5 FABP2 MMP10 CA6 GBA1 EPHB2 AR BMP2K TAOK1 MPG ESR2 CSNK2A1 Alox5 AAK1 PCSK7 FDPS FKBP1A CYP24A1 PRKCA SLC16A1 Sphk2 PIM2 CES1 ITGB5 STK17B SI CFD CYP3A4 TUBB4A ITGAL TUBB1 PDE4A Fkbp1a CYP1B1 GRIA2 ALPL COMT PRKAG1 RPS6KA3 SPHK2 PRSS1 PTGIS MDM4 FBA1 HDAC6 PSMB9 CHRNA7 PDE3B CBR1 RPS6KB1 SOAT2 TOP1 CDC25A RXRB |
| 6 | Oxidant | 162 | ERCC5 DHCR24 AIFM1 CYCS LPO SETD2 SENP1 CAPN3 TIA1 SOD2 BAK1 SHC1 ACTR8 GPX7 RPS23 UCHL1 HMOX2 SIRT6 PON1 FOXC1 VWA8 COQ9 MIR34B MUC1 GCLM ATP6 FUS STIM1 POLG MFN2 CLP1 RYR1 MEAK7 LRRK2 PRDX1 CHGA TP73 MGST1 FXN ACTR5 NOL3 HUWE1 BTK ATP7B SLC19A3 FTH1 CDADC1 TRP-AGG2-5 JUND STC1 SLX1A-SULT1A3 SLC2A10 LDLR PARK7 SGK1 RCAN1 XRCC3 FAM120A CACUL1 ORAI1 NF1 DUSP1 RAD51C ATXN3 OXR1 GRPEL2 STK3 BAD ATM ERCC8 GPX4 SNORD44 SPATS2L POR SHC3 PRXL2A FOXO1 GPT TXNIP OSGIN2 ANXA5 ADPRS DGKK TBC1D24 C10orf88 PGAM5 PRDX3 SIRT7 TFAM DDIT4 DSCAM-AS1 FOXO4 OSGIN1 CYGB SOD1 MIR200A SERPINB1 NDUFV1 ARHI1 IFNG FOXL2 SCARA3 PPIF TNFAIP8 STK4 ABCD1 NFE2L1 COQ8A UBAP2L BHLHE40 EGLN2 VSX1 GPX8 TMEM161A GLRX MSH2 ERCC6 PRDX2 ADIPOR1 SIRT3 KLHDC10 MSRA SDHC SLC25A10 VTRNA1-1 BACH1 CSN1S1 ENTPD1 COA8 TTC23L OXSR1 DLD CAT GSTO1 ADIPOR2 DMD FDXR SELENON NNT SHOX SHC4 TXN TXN2 COL8A2 PDSS2 FOXO3 GPX1 PYCR2 RECQL4 ACOX2 BACH2 MT2A TXNDC17 KCNN4 SESN2 EPO BAX TRP-AGG2-6 SLC17A5 COQ2 SMYD1 PNKD |
| 7 | Inflammation | 245 | CD44 MEFV BID IGF1 MIR146A GAS5 RAC1 ILRUN GDF15 DAZAP1 DELEC1 MIR451A CD4 TUG1 CD1D UBA1 RC3H1 GJA1 NMNAT1 SLC39A14 NFATC1 SNORD15A NPPB EZH2 F2RL1 SEMA3A BTRC MIR223 TNFSF10 HAS2-AS1 MIR193A C3AR1 IL24 TLX1NB MBTPS1 TNFSF15 TERC CELF6 MIRLET7C CCL7 SERPINA1 PRKAA1 IL1RL1 LTBR CXCL8 EGR1 CCN6 IL27RA SMAD5-AS1 CCL27 IL17A BMP6 TBX21 PPARGC1A TNFAIP6 NCOA3 DEFB4A CBLB CTSB ZC3H12A LCK MAP3K8 IL23A C5 IL1A LEF1-AS1 TNFAIP8L3 LPCAT2 CSF3 CCL20 MIR6873 SPP1 IL22 LINC02605 MAD1L1 BMP7 IRAK3 AGER IL21R ELF4 TLR5 CDC42 IL18 CYP2E1 MSR1 SEMA7A IL15 TIE1 TAC1 LRRC19 ACE2 FBXO3 CAMP ICOSLG SELENOS PSMB8 IL3 TGFB1 ACKR2 MYC SOCS3 MST1 MIAT FBN1 S100A8 RPLP2 ZBP1 IL13 MIR21 TNFAIP3 IL12A IGAN1 MIR125A IL27 TNFRSF25 PPT2-EGFL8 PSMD14 PWAR1 ANGPT2 PPP1CA CPA6 ITGAM FASLG SNORD95 GSEC NMUR1 GGT1 SIGIRR IDO1 IL1RAPL2 LINC01554 INS ABHD5 MYD88 MIR145 CCR6 RBCK1 MT-RNR2 MIR30B IL2RA PLCG2 C9orf72 CRH IL10 PGF CUL5 NUBP1 RIPK3 SPI1 ACKR1 DANCR CXCR3 IL3RA IL2 FADD IL17F SELPLG MIR142 FGF2 XIST CXCL12 TET2 RPA1 NR5A2 RIPK1 C5AR2 CXCL9 F2R MIR191 ABCB4 TNIP2 ARPC1B PLA2G7 GRN SOCS1 MIR155 SOX2-OT ALPI P2RX1 MIR25 CAPN5 IL1RN EIF2AK4 MAFB LOC654780 CXCL10 TSLP CCL5 SFTPA2 LINC-ROR CARMN IL33 NLRP1 FLG MEG3 MIR146B HLA-B ATXN1 ELAVL1 SHARPIN CFB MIR199A1 OTULIN NMU SNHG1 MALAT1 OIP5-AS1 CLEC4A ICOS IL1B LPCAT3 WIPF1 IL12B IL9 MIR381 RNF114 NFKBIA BECN1 LGALS8 HAMP IL21 TREM1 YARS1 APOA1 SCN10A CX3CR1 LTA PVT1 CRLF2 CASP4 CEBPB METTL3 CCL3 ATG16L1 TNFSF14 PSMB10 NOTCH1 IL1R1 CD40LG HOTTIP SNHG29 THORLNC HMGB1 S100A9 IL11RA |

**Supplementary table S4** Target protein of top 7 compounds from a combination of *GM-CC-AE* as 10:15:5

| **No.** | **Compounds** | | | | | | | % | Targets | Target proteins |
| --- | --- | --- | --- | --- | --- | --- | --- | --- | --- | --- |
|  | 8HA | CA | DCM | AM | PA | ISA | PDA |  |  |  |
| 1 | ✓ | ✓ | ✓ | ✓ | ✓ | ✓ | ✓ | 53.64 | 4 | CCR1 MAPK1 NFKB1 PTGS1 |
| 2 | ✓ | ✓ | ✓ | - | ✓ | ✓ | ✓ | 39.5 | 18 | CLK4 SLC6A5 GRIN1 KLF5 CDC25C PTPN2 NFE2L2 AURKB SCN3A RORB PIK3R1 FPR2 ADAM10 NR1I2 CTSD APEX1 CYP3A4 TRIM24 |
| 3 | - | ✓ | ✓ | ✓ | ✓ | ✓ | ✓ | 45.05 | 1 | ALOX12 |
| 4 | ✓ | ✓ | ✓ | - | ✓ | ✓ | - | 37.02 | 1 | PSMB1 |
| 5 | ✓ | ✓ | ✓ | - | - | ✓ | ✓ | 38.4 | 2 | AKR1B1 ITK |
| 6 | - | ✓ | ✓ | - | ✓ | ✓ | ✓ | 30.91 | 4 | GLRA1 GPR35 DPP9 DPP8 |
| 7 | ✓ | ✓ | - | - | ✓ | ✓ | ✓ | 15.06 | 12 | CASP6 PTGER2 NR4A1 CASP8 SCN4A NTSR2 TLR4 CDK5 S1PR5 CSNK2B ABCC1 FPR1 |
| 8 | - | ✓ | - | ✓ | ✓ | ✓ | ✓ | 20.61 | 1 | PRKCA |
| 9 | ✓ | - | ✓ | ✓ | ✓ | ✓ | - | 50.06 | 1 | IMPDH2 |
| 10 | ✓ | - | ✓ | - | ✓ | ✓ | ✓ | 38.4 | 8 | HMGCR PTGER3 KLK7 PTGDR PLA2G2A CMA1 PTGER4 LTB4R |
| 11 | - | - | ✓ | ✓ | ✓ | ✓ | ✓ | 43.95 | 4 | PTPN1 PTGES ALOX15 DRD2 |
| 12 | ✓ | - | - | ✓ | ✓ | ✓ | ✓ | 28.1 | 7 | APH1A PPARG PTGS2 PSENEN NCSTN PSEN1 PSEN2 |
| 13 | ✓ | ✓ | ✓ | ✓ | - | - | - | 48.27 | 3 | SRC CDK2 KDM1A |
| 14 | ✓ | ✓ | ✓ | - | ✓ | - | - | 35.23 | 2 | TOP2A NTRK3 |
| 15 | ✓ | ✓ | ✓ | - | - | - | ✓ | 36.61 | 2 | MMP2 MMP9 |
| 16 | - | ✓ | ✓ | ✓ | ✓ | - | - | 40.78 | 1 | ALOX5 |
| 17 | - | ✓ | ✓ | ✓ | - | - | ✓ | 42.16 | 1 | ACP1 |
| 18 | - | ✓ | ✓ | - | ✓ | - | ✓ | 29.12 | 1 | KCNK2 |
| 19 | - | ✓ | ✓ | - | - | ✓ | ✓ | 29.81 | 3 | AKR1B10 CA2 CA1 |
| 20 | ✓ | ✓ | - | - | ✓ | ✓ | - | 12.58 | 1 | MAP2K2 |
| 21 | ✓ | ✓ | - | - | ✓ | - | ✓ | 13.27 | 2 | PTPN11 CDK1 |
| 22 | - | ✓ | - | - | ✓ | ✓ | ✓ | 6.47 | 4 | KDM4C LOX1.1 POLB TDP1 |
| 23 | ✓ | - | ✓ | ✓ | - | ✓ | - | 48.96 | 1 | HIF1A |
| 24 | ✓ | - | ✓ | - | ✓ | ✓ | - | 35.92 | 1 | MAPK14 |
| 25 | - | - | ✓ | ✓ | - | ✓ | ✓ | 42.85 | 3 | PLK1 ABL1 CYP19A1 |
| 26 | - | - | ✓ | - | ✓ | ✓ | ✓ | 29.81 | 13 | HSD17B3 KCNK9 AKR1C2 CHRNB4 CYP26B1 CYP2A6 DPP7 TRPA1 HSD11B1 PIK3CD RXFP1 PDE4A MDM4 |
| 27 | ✓ | - | - | ✓ | ✓ | - | ✓ | 26.31 | 1 | APH1B |
| 28 | ✓ | - | - | ✓ | - | ✓ | ✓ | 27 | 1 | PRSS1 |
| 29 | ✓ | - | - | - | ✓ | ✓ | ✓ | 13.96 | 14 | CYP26A1 PPARA GPR55 CYSLTR2 SCN2A SLC40A1 EDNRA TBXA2R PTGER1 TBXAS1 KEAP1 FFAR1 PTGDR2 PSMB9 |
| 30 | - | - | - | ✓ | ✓ | ✓ | ✓ | 19.51 | 1 | TLR2 |
| 31 | ✓ | ✓ | ✓ | - | - | - | - | 34.13 | 14 | PRCP MMP13 BACE1 C5AR1 PYGL HTR2C CACNA1B METAP2 PDGFRB ACACB PDGFRA MIF MMP1 GRIA2 |
| 32 | - | ✓ | ✓ | ✓ | - | - | - | 39.68 | 7 | GLO1 IKBKB MAOA ABCB1 CHUK PDE4D APP |
| 33 | - | ✓ | ✓ | - | ✓ | - | - | 26.64 | 2 | PTPN22 STAT1 |
| 34 | - | ✓ | ✓ | - | - | ✓ | - | 27.33 | 6 | PIK3CG AOC3 CCR2 PSMB2 QRFPR SLC2A1 |
| 35 | - | ✓ | ✓ | - | - | - | ✓ | 28.02 | 1 | NR0B2 |
| 36 | ✓ | ✓ | - | ✓ | - | - | - | 23.83 | 2 | CFTR CASP1 |
| 37 | ✓ | ✓ | - | - | ✓ | - | - | 10.79 | 2 | SLC9A1 MME |
| 38 | ✓ | ✓ | - | - | - | ✓ | - | 11.48 | 3 | NR3C2 SAE1 CASP7 |
| 39 | ✓ | ✓ | - | - | - | - | ✓ | 12.17 | 1 | MMP12 |
| 40 | - | ✓ | - | ✓ | - | - | ✓ | 17.72 | 2 | PRKCD Alox5 |
| 41 | - | ✓ | - | - | ✓ | ✓ | - | 3.99 | 2 | KDM6B GBA1 |
| 42 | - | ✓ | - | - | ✓ |  | ✓ | 4.68 | 9 | MGLL CDC25B PIN1 ICAM1 CAPN1 AHCY TLR8 ITGB2 ITGAL |
| 43 | - | ✓ | - | - | - | ✓ | ✓ | 5.37 | 8 | GSR NAALAD2 SELL FOLH1 ENPEP THRA SELP ACLY |
| 44 | ✓ | - | ✓ | ✓ | - | - | - | 47.17 | 3 | ITGB1 IMPDH1 AGTR1 |
| 45 | ✓ | - | ✓ | - | ✓ | - | - | 34.13 | 2 | NOS2 SCD |
| 46 | - | - | ✓ | ✓ | - | ✓ | - | 40.37 | 2 | MTOR HDAC1 |
| 47 | - | - | ✓ | ✓ | - | - | ✓ | 41.06 | 1 | AKT1 |
| 48 | - | - | ✓ | - | ✓ | - | ✓ | 28.02 | 2 | DNM1 TRPV1 |
| 49 | - | - | ✓ | - | - | ✓ | ✓ | 28.71 | 7 | NAMPT NR1H4 CA3 AKR1C3 GABRA2 PTPN7 LTA4H |
| 50 | ✓ | - | - | ✓ | - | ✓ | - | 24.52 | 1 | OPRK1 |
| 51 | ✓ | - | - | - | ✓ | ✓ | - | 11.48 | 1 | MDM2 |
| 52 | ✓ | - | - | - | ✓ | - | ✓ | 12.17 | 2 | CNR2 TACR2 |
| 53 | ✓ | - | - | - | - | ✓ | ✓ | 12.86 | 5 | TMPRSS6 FNTA BLM FNTB CHRNA7 |
| 54 | - | - | - | ✓ | ✓ |  | ✓ | 17.72 | 2 | fabG PLCG1 |
| 55 | - | - | - | ✓ |  | ✓ | ✓ | 18.41 | 3 | RARA THRB BCL2L1 |
| 56 | - | - | - | - | ✓ | ✓ | ✓ | 5.37 | 55 | PPARD KDM4A BBOX1 PAM PLA2G2C SLC6A11 HTR2B HSD11B2 RBP4 MAP3K5 CTSG KDM5C PLA2G5 CES2 FFAR4 DCUN1D1 NR2E3 SLCO2A1 FABP5 HAO1 PIK3CB SLC22A6 KDM5A KDM2A hdaH SLC25A20 ANPEP OXER1 GABRQ PTGFR PDE4B FAAH GABBR1 SLC22A8 FABP3 DAGLA PLA2G4A GGPS1 cqsS FABP4 CPT1B luxP P2RY12 GABBR2 COL4A3BP Jhe FABP2 PLA2G10 SLC16A1 CES1 EPHX2 S1PR3 TOP1 CDC25A RXRB |
| 57 | - | ✓ | ✓ | - | - | - | - | 25.54 | 19 | TNIK FLT1 TTR CA14 DNMT1 GUSB GLS ADRB1 IKBKG CA5B AKR1C4 CA9 CA5A PDE3A PLK4 CA6 CA12 TUBB1 CA7 |
| 58 | ✓ | ✓ | - | - | - | - | - | 9.69 | 12 | ACE KDR F13A1 MMP8 ELANE MAPK8 CHRM1 TFPI MARK4 FPRL2 AMPD3 CASP3 |
| 59 | - | ✓ | - | ✓ | - | - | - | 15.24 | 3 | Akr1b1 SERPINE1 EGLN1 |
| 60 | - | ✓ | - | - | - | ✓ | - | 2.89 | 2 | GNPAT DOT1L |
| 61 | - | ✓ | - | - | - | - | ✓ | 3.58 | 1 | ADORA3 |
| 62 | ✓ | - | ✓ | - | - | - | - | 33.03 | 7 | HDAC4 EGFR HTR7 PDE7A AURKA STAT3 CYP2C9 |
| 63 | - | - | ✓ | ✓ | - | - | - | 38.58 | 15 | PLAU ABCG2 CA13 AXL XPO1 BCHE DRD3 RELA ODC1 PNLIP MELK ACHE GSK3B CYP1B1 HSP90AA1 |
| 64 | - | - | ✓ | - | ✓ | - | - | 25.54 | 3 | GRM2 PTPN6 PRKAB1 |
| 65 | - | - | ✓ | - | - | ✓ | - | 26.23 | 7 | CHRNA1 HDAC8 HDAC2 HPSE P2RX7 HDAC6 PDE3B |
| 66 | - | - | ✓ | - | - | - | ✓ | 26.92 | 2 | KLK5 GABRG2 |
| 67 | ✓ | - | - | ✓ | - | - | - | 22.73 | 4 | DUSP3 TNF CTNNB1 ITGA4 |
| 68 | ✓ | - | - | - | ✓ | - | - | 9.69 | 5 | MC4R NR3C1 DGAT1 CCKBR ESR2 |
| 69 | ✓ | - | - | - | - | ✓ | - | 10.38 | 4 | CPA1 KIF11 OPRD1 WDR5 |
| 70 | - | - | - | ✓ | ✓ | - | - | 15.24 | 2 | PRKCH SQLE |
| 71 | - | - | - | ✓ | - | ✓ | - | 15.93 | 4 | REST DHFR CCR5 IDH1 |
| 72 | - | - | - | ✓ | - | - | ✓ | 16.62 | 1 | GPR84 |
| 73 | - | - | - | - | ✓ | ✓ | - | 2.89 | 7 | NR1H3 ADRA2B HNF4A PGR SLC22A12 SLC6A13 METAP1 |
| 74 | - | - | - | - | ✓ | - | ✓ | 3.58 | 35 | POLK ERAP2 POLI LPAR3 LPAR4 LNPEP FABP1 S1PR1 GPR34 EPHX1 lpxC FabI Slco2b1 TERT EST1 P2RY10 CNR1 DPEP1 POLH ACER2 LPAR6 CPT1 S1PR4 SPHK1 PLA2G4C GPR174 PLA2G4B ENPP2 LPAR1 fabZ PTGIR lasR LPAR2 ACAT SOAT2 |
| 75 | - | - | - | - | - | ✓ | ✓ | 4.27 | 38 | VDR RXRG glmU HSD17B10 CACNA2D1 SERPINA6 RORA pepN KDM7A S1PR2 RXRA UGT2B7 Slc22a20 NOD1 NPC1L1 BHMT CYP4F2 GSTK1 GPBAR1 SHBG GRM5 G6PD ABAT GR3 CDC45 GABRR1 RARB PHF8 PTGES2 PLG RARG CYP4A4 Gabbr2 murF Gabrg1 AR CSNK2A1 PTPRC |
| 76 | - | ✓ | - | - | - | - | - | 1.1 | 61 | SLC37A4 Mmp2 XDH NEU2 MGAM KDM4D CHRM5 PTK2B Ca13 Polb GRK6 FPGS GRK5 FTO SUV39H1 MMP14 HSPD1 LGALS3 pol MMP16 NEU4 HK2 DNMT3B Gsk3b IGF1R HPRT1 GRIK2 sssIM Ptgs2 ERAP1 FBP1 MAPT INMT GRIK3 TREH KMT2A CHRM2 ECE1 CHRM4 PDE9A SLC13A5 INSR YARS OGA Hsd17b3 NEU3 aroB gag-pol AMY2A P2RX4 HK1 CHRM3 IGFBP3 PYGM PDE1B ENGASE CASP2 ADAMTS5 ADA SI SMYD2 |
| 77 | - | - | ✓ | - | - | - | - | 24.44 | 129 | ERBB4 ZAP70 MST1R PDF PARP15 GRM3 EP300 NTSR1 HTR1A FGR ND1 MET MTNR1B ALOX15B NUS1 MAOB CYP1A2 TAOK3 LYN ITGA3 ACVRL1 PTPN12 CYP2C19 BMPR1A LGMN CA4 GPR183 ROCK2 TEK ACR HTR1B ACAT1 CYP27B1 AVPR1B HDAC5 SLC1A1 DDIT3 PTK2 FOS KCNA3 AGPAT2 MCOLN3 RASD2 HCRTR2 TIMM23 GCK ERO1A NRG3 SND1 F9 ACVR1 ESRRA ATR CYP1A1 KRAS DRD4 CTDSP1 PAX8 GLA NQO2 RET GSTP1 HDAC11 ADRA1A HSF1 PDE10A PARP14 XBP1 AHR HTR1D GAA GFER GABRB3 BRD4 ACVR1B SLC1A3 PTAFR NOX1 TGFBR2 TLR9 RAD51 HCRTR1 DYRK1A SENP6 TUBA1A MPO JAK3 ADRA1B JAK1 ENPP1 JAK2 TNFRSF1A ERVW-4 APOBEC3G CISD1 EIF4H APOBEC3A OLR1 BMPR1B MCHR1 UTS2R ASF1A PRKDC BMP4 GAK PTK6 NOD2 F3 GRM1 SNCA AKR1A1 PTPA ADRA1D ERN1 JUN CSF1R EPHB2 TAOK1 AAK1 MTNR1A CYP24A1 MCL1 STK17B CFD TUBB4A TRPM5 KARS NR2F2 RPS6KB1 |
| 78 | ✓ | - | - | - | - | - | - | 8.59 | 47 | NPY5R MKNK2 MMP3 FGFR1 GALK1 NLRP3 ITGA2B ACACA TNK1 DPP4 ADORA1 SYK ADH5 P2RX3 TTL PDE6D PDE5A GZMB ADK MAPK10 APLNR CDK5R1 ITGB7 OPRM1 CASP9 CACNA1H CREBBP ITGB6 F5 ITGAV EDNRB SLC5A1 HCAR2 FDFT1 SLC5A2 ULK3 STING1 ITGB3 CTSA AMPD2 MMP10 BMP2K MMEL1 SOAT1 FKBP1A ITGB5 PTGIS |
| 79 | - | - | - | ✓ | - | - | - | 14.14 | 62 | PRKCE HSD17B1 PRKCG PTPRS CXCR1 ARG PSMG3 FASN PRKCB CGAS TLR1 NUDT1 NQO1 PIK3CA ERBB2 CREB1 PRKCI PPO2 CHEK1 CELA1 SYN1 KCNH2 CXCR2 F10 LDHB TNNI3 CTSV MAPK3 HSP90AB1 HSP90B1 CHEK2 CALCA TNNC1 TCF4 KISS1R KLKB1 TYR nanH Abcb1a PDK1 KLK2 LDHA TNNT2 F2 KLK1 MAP2K1 SIRT1 CNOT7 GCGR CCND1 CAN2 CDK4 STAT6 MPG VCP PCSK7 ADCYAP1R1 IL6 COMT RPS6KA3 CBR1 CCR4 |
| 80 | - | - | - | - | ✓ | - | - | 1.1 | 27 | CACNA1C PRKAA2 TRPM8 SMO PDCD4 CYSLTR1 psbA PAFAH1B2 Lef1 GAPG MC5R GABRG1 LOX1.4 TSPAN1 lipN STS ABCC2 GCG NR1H2 PRKACA RORC ESR1 MC1R KAT5 PIM2 PRKAG1 SPHK2 |
| 81 | - | - | - | - | - | ✓ | - | 1.79 | 23 | GGH SLC6A4 Grm4 GRM8 P2RY6 PLAT SIRT5 Grm6 RNPEP SLCO2B1Ã‚Â  ST14 Folh1 HDAC3 Grm8 GRM6 PHGDH NMT1 CAMKK2 HDAC10 FUT7 GSP CHKA NS |
| 82 | - | - | - | - | - | - | ✓ | 2.48 | 78 | PRE7 GSTA1 CPT1BÃ‚Â  HPD Dusp6 GNAI3 agrA Slc6a13 Prkch luxR MEP2 SLC22A2 GSTM1 Lss ALOX5AP PLA2G1B PTGER1Ã‚Â  Sqle GNAI1 POLL SRD5A1 Abcc2 NAAA FPPS POLM PRE2 CCKAR LPAR5 ICL1 PAOX Ces2c Slc6a11 ATG4B Naaa ADH1A ASAH1 blaIMP-1 SMPD2 resT EcR Fasn GNAO1 SPPS ARG2 SLC22A1 Slc6a8 Cdc25b OR51E2 traR Prep ADH7 AKR1C1 ADH1B fbpC Gpr34 GABRB2 MPEG1 LAP3 HK PLB1 ADH1C phzR CPT1A CSNK2A2 GBA DNTT LMNA plc MT-ND1 Msr1 FDPS dxs Sphk2 Fkbp1a ALPL CPT2 FBA1 KDM4E |

**Supplementary table S5** The overlap between the top 7 compounds in combination of *GM-CC-AE* as 10:15:5 and target proteins of inflammation and oxidative stress

| No. | Targets | Total | CA | DCM | 8HA | AM | PA | ISA | PDA |
| --- | --- | --- | --- | --- | --- | --- | --- | --- | --- |
| 1. | XDH | 1 | ✓ |  |  |  |  |  |  |
| 2. | ALOX5 | 5 | ✓ | ✓ |  | ✓ | ✓ |  | ✓ |
| 3. | ELANE | 2 | ✓ |  | ✓ |  |  |  |  |
| 4. | NFE2L2 | 6 | ✓ | ✓ | ✓ |  | ✓ | ✓ | ✓ |
| 5. | TNF | 2 |  |  | ✓ | ✓ |  |  |  |
| 6. | MPO | 1 |  | ✓ |  |  |  |  |  |
| 7. | CASP1 | 3 | ✓ |  | ✓ | ✓ |  |  |  |
| 8. | TRPA1 | 4 |  | ✓ |  |  | ✓ | ✓ | ✓ |
| 9. | PTGS2 | 6 | ✓ |  | ✓ | ✓ | ✓ | ✓ | ✓ |
| 10. | OLR1 | 1 |  | ✓ |  |  |  |  |  |
| 11. | IL6 | 1 |  |  |  | ✓ |  |  |  |
| 12. | MAPK1 | 7 | ✓ | ✓ | ✓ | ✓ | ✓ | ✓ | ✓ |
| 13. | FOS | 1 |  | ✓ |  |  |  |  |  |
| 14. | MAPK8 | 2 | ✓ |  | ✓ |  |  |  |  |
| 15. | ICAM1 | 3 | ✓ |  |  |  | ✓ |  | ✓ |
| 16. | MAPK3 | 1 |  |  |  | ✓ |  |  |  |
| 17. | SIRT1 | 1 |  |  |  | ✓ |  |  |  |
| 18. | PTGS1 | 7 | ✓ | ✓ | ✓ | ✓ | ✓ | ✓ | ✓ |
| 19. | TLR2 | 4 |  |  |  | ✓ | ✓ | ✓ | ✓ |
| 20. | JUN | 1 |  | ✓ |  |  |  |  |  |

**Supplementary table S6** The list of genes of Thai herbal combination of *GM-CC-AE* as 5:10:15, inflammation and oxidative stress.

| NO. | Names | Total | Gene targets |
| --- | --- | --- | --- |
| 1. | (5-10-15)-Inflammation-oxidant | 23 | XDH ALOX5 ELANE SELE NFE2L2 TNF MPO CASP1 TRPA1 PTGS2 OLR1 IL6 PARP1 MAPK1 FOS MAPK8 ICAM1 MAPK3 TP53 SIRT1 PTGS1 TLR2 JUN |
| 2. | (5-10-15)-Oxidant | 34 | ERCC5 SENP1 PRKCG NOS2 UCHL1 REST CTSV RYR1 LRRK2 MAPT BLM CTNNB1 STK3 ATM SNCA KEAP1 VCP ACHE CASP3 ACE GSR BACE1 FABP1 MDM2 SLC1A1 NDUFV1 G6PD IGF1R ERO1A STK4 AKR1B1 SIRT3 RAD51 APP |
| 3. | (5-10-15)-Inflammation | 85 | MMP2 PLAU EP300 NLRP3 HSD11B2 NR1H3 IMPDH2 DPP4 EZH2 CXCR2 GRIK2 CASP8 PPARG PRKAA1 MAPK14 CTSB LCK MAP3K8 IKBKG CCR2 CREBBP GGPS1 ITGB6 SELP TLR8 ITGAV JAK2 CHUK TNFRSF1A STAT3 F2 CSNK2B STING1 MSR1 ESR1 ACE2 FBXO3 PSMB8 GSK3B MMP1 STAT1 MMP12 PTGER4 TRPV1 VDR NR3C2 IDO1 ABCG2 CCR6 PLCG2 NAMPT NOD1 PIK3CG EGFR IKBKB CXCR3 IL2 SYK RPA1 F10 F2R HPRT1 RELA HIF1A CFTR PTPN6 P2RX1 ALPI XBP1 DPEP1 TLR4 TLR9 JAK1 FABP4 AKT1 CCR5 MIF NFKB1 NOD2 SCN10A P2RX7 PSMB10 LTA4H S1PR3 MMP9 |
| 4. | Inflammation-oxidant | 28 | CERNA3 PINK1 MIR7-3HG BDNF-AS HMCN1 CFH CRP MIR29B1 TRA-TGC7-1 H19 MIR141 TRPM2 VEGFA TIMP1 CCL2 HMOX1 PRKN VNN1 CAV1 MIR34C HFE PKM SOD2-OT1 TMX2-CTNND1 LINC01672 ALB SCARNA5 ADIPOQ |
| 5. | (5-10-15) | 1161 | PTGER3 ERBB4 KCNJ2 UGCG ZAP70 PRCP SLC6A2 PDE6A BBOX1 NDUFS6 CYP26A1 PTPRCAP NDUFB1 NMBR TNIK PDF CCNA1 HSD17B1 QARS CLK4 CXCR4 MPL GSTA1 CYBA NAALAD2 HSD17B2 HSD17B10 POLK PAK1 KLK14 NTRK1 PLA2G2C SLC6A11 RPL19 MMP7 MITF NTSR1 GYS1 CA13 MMP13 CACNA2D1 CCNB1 CDK14 SLC6A4 NDUFS1 POLI NDUFA6 KCNK3 NDUFAF3 GNAI3 FGR NDUFAF4 ND1 STK16 NDUFAB1 CHRM5 FGFR1 GALK1 SERPINA6 MET MTNR1B PTK2B MAP3K3 CASP6 GRM2 NR4A2 SRPK1 FADS1 SLC22A2 RPS6KA5 MAPKAPK2 FASN PHLPP2 TRPM8 PTPN22 TAOK3 ITGA3 RIOK1 AKR1B10 CDK15 CDC25B CYP2D6 LPAR4 PSMA3 PTPN12 ACVRL1 S1PR2 TGM2 SLK MECP2 PSMA1 STK26 PRKCB MAP3K5 RXRA PDLIM7 C5AR1 CYP2C19 NOS1 TBK1 CA14 BMPR1A SLCO2B1 ROCK2 ACACA VKORC1 TLR1 TEK UGT2B7 EED CCR9 SLC8A1 GRK5 HES1 AP1S2 ACAT1 NUDT1 SLC18A3 GRK1 PLK3 PTPN11 KDM5C SSTR2 PTGDR CES2 SELL PLCG1 PLK1 NDUFA11 HTR2C ST3GAL1 MKNK1 GPR55 MDH1 NEK4 RBBP9 NCOR2 HTR7 CD38 DRD5 PSMC2 CCNE1 BHMT AVPR1B PLA2G2A FOLH1 HRH4 TMIGD3 ALK MAOA MINK1 HDAC5 ALDH2 NR2E3 CDK8 FAS HDAC7 GRB2 PIN1 SLCO2A1 PRKCI CDK1 PTK2 NDUFS8 CYSLTR2 ADH5 NDUFA8 SHBG FABP5 DNM1 P2RX3 SORT1 PREP CHEK1 BCHE RASD2 KIF11 BCL2 TAS1R3 HCRTR2 MMP16 PSMB6 MAP3K14 SCP2 SLC40A1 DRD3 ND3 BDKRB1 HAO1 PDE6D POLM PRKG2 NDUFS4 PSMD3 PIK3CB CLK2 SLC22A6 NPM1 GCK THRB CELA1 PSMD11 LARS F7 ADORA2A EDNRA SYN1 KCNH2 PDE4C CAMK2D SND1 F9 LIPE B4GALT1 ESRRA SRPK3 ATR CYP1A1 LDHB PIP4K2B TNNI3 TACR1 CAPN1 NR0B1 CDK17 NDUFA2 GNRHR SENP7 HTR3A OXTR CDC25C CYP11B2 KDM5A MAPK10 CS KDM2A CACNB1 SCN4A PTPRB EZR IL5 KRAS PTGES NOTUM ERAP1 GIP ATG4B OPRK1 TKTL1 CHRM1 CCNB3 CHRNB2 MERTK APLNR CRPPA TBXA2R ALOX15 NDUFC1 CACNA1B DMPK GLA SLC1A5 RARB CALCA PRKCD NDUFB4 NQO2 MARK3 NEU1 TMPRSS6 AKR1C2 TNNC1 HCAR3 RET GSTP1 GABRQ TCF4 CDY1 GABRG1 AHCY NRAS AURKA MAPK9 SRD5A2 ADRA1A ASAH1 PTPN2 RIPK2 CNGA1 GIPR CHRNB4 GLP1R HDAC9 PARP14 PTGFR KISS1R PTGER1 SMPD2 PSMC6 ABCB1 CNR1 AHR CBX6 DHFR PIM1 UBE2N DYRK2 NR3C1 ITGB7 KLKB1 GAA F11 MAP2K6 GNAO1 HDAC3 RPS6KA6 FAAH ECE1 HCK PPP1CC TARS PNLIP AURKB MAP4K1 CDCA7L MTOR GABBR1 CHRM4 SCN3A NUAK1 CYP2A6 NDUFS2 NDUFA4L2 KDM1A BRD4 NDUFA13 CYP19A1 SLC22A8 SLC22A1 HRH2 AOC3 FABP3 CRABP1 SLC27A1 TRPV2 CAMKK1 RORB CDK6 PDGFRB ADRB1 CSK CTSS TNKS2 SIRT2 NDUFB8 RAF1 ADH7 CMA1 CACNA1H FYN HCRTR1 TLK1 PLA2G4A SENP6 ITGB1 DRD2 AKR1C4 DSTYK MAP2K2 PSMC4 DBT ABCC2 TUBA1A ND6 PSMB2 KLK2 ADRA1B MPI P4HTM UQCRB PSMD8 FGFR2 PIP5K1A AOC1 AMY2A KIT FNTB ADAMTS4 GABRB2 LDHA EDNRB TACR2 EGLN1 SCD DPP7 GRIN2B DGAT1 PDPK1 S1PR4 SENP8 CPT1B ERVW-4 SPHK1 PLA2G4C TNNT2 MYLK4 GRIA1 CA9 HCAR2 TBXAS1 GPR174 TNK2 NDUFA4 FDFT1 MT-ND4 LGALS4 APOBEC3A SQLE PSENEN TAOK2 ND4 ADAM10 MAP4K3 NDUFB5 CLK3 ADH1C PAK3 CHN2 TGFBR1 P2RY12 SLC6A13 CYP2C9 HK1 CNOT7 GRK2 NT5E NCSTN UTS2R MCHR1 GCGR CCND1 CA5A CTSK SGK2 TNKS PRKDC PSMD2 PSMD12 CDY1B CAN2 ACLY STK33 PGGT1B DAPK2 PFKFB3 PRF1 MAPK7 BMP4 CDK4 AGTR1 TYMS GABRA2 MAN2A1 PIM3 CSNK2A2 GBA CBX8 MELK GRM1 SSTR3 PDE4D ITK PPIA CSNK1D CASP7 CDK16 HRAS PER2 PRKACB NDUFAF1 PDE3A CCKBR MAP2K4 NDUFB6 ADRB2 CPB1 BCL6 MDH2 MAP3K6 DHCR7 CTSD HPGD STAT6 ENPP2 RIOK3 PIK3CD AREG AP2S1 MME CDKL2 APEX1 GRIN2A PTPA PSEN2 QPCT ADRA1D KAT6A SGSM3 AVPR2 PTGIR NTRK3 PLK4 RXFP1 CCNA2 TYRO3 FANCF CCNC MMEL1 RPS6KB2 ADRM1 CHKA BUB1 SOAT1 FFAR1 SP4 MAP4K5 PIP5K1C PLAA PLA2G10 NDUFV2 SIGMAR1 MTNR1A PDK4 MCL1 PSMA5 PRNP PSMC1 AMPD3 ADCYAP1R1 CA12 DOT1L CBX2 EIF2AK3 GPR88 TRPM5 POLB EPHX2 PTPN7 LPAR2 KARS ANGPT1 GABRA1 CDC7 CPT2 PTGDR2 SLC2A1 TAS2R14 KCNQ1 LSS LIMK2 PRLHR WEE1 NR2F2 ADCY1 BCL2A1 RPS6KA2 EBP SLC22A3 SLC22A20P LTB4R TDP1 HSP90AA1 NDUFC2 DYRK1B TRIM24 PROC GBA2 NDUFB3 CACNA2D2 CA7 FPR1 PTPRC CCR4 CLK1 KDM4E PKN2 ITGA4 CDK18 HMGCR PPARD KDM4A PSMA2 NPY5R PRKCE PSMA4 ROCK1 STK10 MST1R CPA1 MGAM NDUFB10 RXRG PRKAA2 CILK1 KDR KCNK2 PAM BRD9 NDUFV3 PARP15 HPD GRM3 MC4R GLO1 TAS2R31 ERAP2 FLT1 HTR1A ALOX12 ND2 RARA CARM1 PTPRS LATS2 MKNK2 PTPN1 YWHAG CBFB FCGRT MMP3 PRKCZ HTR4 CRHR1 PDE2A LEO1 LPAR3 LRP6 AXL CRABP2 PPARA CNR2 F13A1 MGLL CSNK1E RORA ALOX15B CDK9 FAP KCNJ1 CDYL2 DUSP3 KDM7A HRH3 TTR TUBB2B CCR1 CXCR1 HTR2B PSMG3 NUS1 MAOB ITGA2B PTGDS PTGER2 SLC5A7 CYP1A2 MMP8 HDAC4 LYN DUSP6 GLRA1 SLC28A2 RBP4 CBX4 NDUFB7 BRPF1 GSTM1 ADAM33 S100A4 MGAT2 LGMN CA4 GPR183 HSD17B3 PYGL CTSG NPEPPS CHRNA1 PSMB11 ACR PSMB7 ALOX5AP KLK7 DNMT1 HTR1B TNK1 PRKG1 P2RY6 PLA2G1B SLC5A5 RASGRP1 CYP27B1 ADRA2B SUV39H1 SEM1 PLA2G5 TIMM10 MMP14 PLAT NLK KCNK9 NDUFAF2 NPC1L1 ICMT GNAI1 AKT2 POLL NQO1 TYK2 FFAR4 MYLK CYP4F2 EIF4A1 GSTK1 PRKD2 S1PR1 SIRT5 PIK3CA GPBAR1 FLT3 MAP3K2 TSPO CCNB2 ADORA1 NR4A1 DCUN1D1 KDM4C SLC6A5 ERBB2 CREB1 SRPK2 CACNA1A ALDH3A1 DDIT3 LGALS3 SRD5A1 GRIN1 NAAA KCNA3 AGPAT2 APH1A SCN2A CYP27A1 TRHR XPO1 RPS6KA4 MCOLN3 LATS1 HTR6 BRD7 TTL GRM5 ENPEP GPR34 PSMD6 LPAR5 TIMM23 CCNT1 HK2 LIPG OPRD1 ELOVL6 KLF5 EPHX1 PAOX PDE5A TOP2A PRTN3 NPBWR1 AVPR1A FEN1 HDAC8 KLK5 CBX7 NRG3 GPR35 ACVR1 CALCRL PRP4K TRIM54 ABAT ALDH1A1 TAS1R2 MTAP DGAT2 BCL2L1 ADORA3 ABL1 PSMA7 GZMB BRDT SLC16A3 BMP1 SLC6A8 CCNE2 ADK TNNI3K SLC9A1 PDE7A HDAC2 CTSL PLEC PSMB4 CA2 HSP90AB1 TAS1R1 SLC25A20 NDUFA1 SYNE1 HSP90B1 PSMB5 NCOA1 DPP9 FER TRPC6 CDK7 GUSB SORD HNF4A EHMT1 GPR6 CHEK2 RASGRP3 FBP1 DRD4 SUZ12 VARS CDK5R1 CTDSP1 GABRR1 TERT FUCA1 PAX8 HIPK4 SLC2A4 NTSR2 DUT MAP2 NOS3 ANPEP OXER1 ADH1A P2RY10 CYP11B1 CETP HDAC11 PTPRA KCNA5 HSF1 PSMD4 NDUFA7 PDE10A METAP2 SLC29A1 SRC PKN1 CASR NR0B2 CDK2 ACP3 PDE4B PGR CHRM2 GPR84 GRK4 PPP2CA NR1H4 ODC1 SSTR1 ADORA2B PDE11A PHF8 TFPI HTR1D POLH THRA MAPK11 PTGES2 OPRM1 ACER2 DPP8 CA3 CYP26B1 NAB1 GFER NADK GABRB3 MAN1B1 GPR17 PLG TYR nanH SERPINE1 EIF2AK1 NDUFB11 SLC13A5 WDR5 Abcb1a RARG GLS STS ACVR1B FFAR2 VCAM1 SLC1A3 PSMD1 PTAFR PORCN NOX1 EWSR1 LPAR6 OR51E2 CTBP2 STK11 INSR PSMC5 TGFBR2 MAP4K2 PDK1 YARS MARK4 FNTA ACP1 CASP9 CIT ALPG ADH1B DAGLA DYRK1A CDK5 BRD2 LPL CA5B RPS6KA1 PRKCQ LIPC JAK3 PSMD13 APH1B ADAM17 PHKG1 GABRG2 FCER2 QRFPR F5 LYZ ATP12A PARP3 S1PR5 ENPP1 NDUFA3 SCNN1A P2RX4 GABRA5 MAPK15 PRKCH PIK3R1 ACACB PDGFRA ADRB3 MPEG1 UPP1 NDUFA12 SLC6A9 PABPC1 APOBEC3G NDUFS5 SLC5A1 CISD1 MLNR EIF4H FPR2 LAP3 IMPDH1 PRKACA ABCB11 SLC22A12 RORC NDUFA9 AURKC IDH1 CAMKK2 TAAR1 NUAK2 CERT1 HSD17B8 PRKAB1 HDAC10 HDAC1 PSMD7 GRM4 KLK1 PLB1 KMO SLC5A2 RAB9A ULK3 MAP2K1 ABCC1 ITGB3 MARS NDUFB2 BMPR1B REN KDM2B GABBR2 ASF1A CTSA CDKL5 PSMC3 FUT5 HPSE TPPP SAE1 ST6GAL1 PSMA8 PSMB3 TDO2 HSD11B1 GSK3A TOP2B AKR1C3 TLK2 NPY4R PSMB1 IGFBP3 CTSH GAK PSMA6 CPT1A PTK6 GRIA4 DNTT F3 MAP4K4 CTSZ ND4L HTR2A GRK7 NR1I2 DLAT LMNA NDUFA10 AMPD2 PYGM FPRL2 AKR1A1 PSEN1 PLA2G4B GPR139 LPAR1 PDK3 SGK3 BRD3 NDUFS3 CA1 GSTM2 ERN1 CSF1R IRAK4 DHODH ADAMTS5 HGFAC MMP10 FABP2 TTK GBA1 CA6 EPHB2 AR BMP2K CHRNG CALM1 CTRB1 TAOK1 FLT4 MPG MAP3K11 ESR2 AGTR2 GABRA3 CYP2J2 CSNK2A1 HSPG2 BRAF AAK1 PCSK7 FDPS SSTR5 AKT3 MAP3K19 FKBP1A CYP24A1 NDUFS7 SSTR4 AMD1 SLC16A1 PRKCA NPC1 SF3B3 PIM2 ITGB5 CES1 EPHB4 GHSR STK17B TYMP HIPK1 SI CFD TUBB4A CYP3A4 TRPC3 CELA2A CDK3 TUBB1 PDE4A PRUNE1 SCN9A MLYCD NDUFA5 CACNA1I ND5 CYP1B1 GRIA2 ALPL COMT PRKAG1 CACNA1G SPHK2 RPS6KA3 ROS1 SLC5A4 PRSS1 PTGIS MDM4 HDAC6 PSMB9 CHRNA7 NDUFB9 PDE3B CBR1 RPS6KB1 SOAT2 PGK1 TOP1 DRD1 CDYL CDC25A CTSC RXRB |
| 6. | Oxidant | 153 | DHCR24 AIFM1 CYCS LPO SETD2 CAPN3 TIA1 SOD2 BAK1 SHC1 ACTR8 GPX7 RPS23 HMOX2 SIRT6 PON1 FOXC1 VWA8 COQ9 MIR34B MUC1 GCLM ATP6 FUS STIM1 POLG MFN2 CLP1 MEAK7 PRDX1 CHGA TP73 MGST1 FXN ACTR5 NOL3 HUWE1 BTK ATP7B SLC19A3 FTH1 CDADC1 TRP-AGG2-5 JUND STC1 SLX1A-SULT1A3 SLC2A10 LDLR PARK7 SGK1 RCAN1 XRCC3 FAM120A CACUL1 ORAI1 NF1 DUSP1 RAD51C ATXN3 OXR1 GRPEL2 BAD ERCC8 GPX4 SNORD44 SPATS2L POR SHC3 PRXL2A FOXO1 GPT TXNIP OSGIN2 ANXA5 ADPRS DGKK TBC1D24 CACNA1C C10orf88 PGAM5 PRDX3 SIRT7 TFAM DDIT4 DSCAM-AS1 FOXO4 OSGIN1 CYGB SOD1 MIR200A SERPINB1 ARHI1 IFNG FOXL2 SCARA3 PPIF TNFAIP8 ABCD1 COQ8A NFE2L1 UBAP2L BHLHE40 EGLN2 VSX1 GPX8 MSH2 GLRX TMEM161A ERCC6 PRDX2 ADIPOR1 KLHDC10 MSRA SDHC SLC25A10 VTRNA1-1 BACH1 CSN1S1 ENTPD1 COA8 TTC23L OXSR1 DLD CAT GSTO1 ADIPOR2 DMD FDXR SELENON NNT SHOX SHC4 TXN TXN2 COL8A2 PDSS2 FOXO3 GPX1 PYCR2 RECQL4 ACOX2 MT2A BACH2 TXNDC17 KCNN4 SESN2 EPO BAX TRP-AGG2-6 SLC17A5 COQ2 SMYD1 PNKD |
| 7. | Inflammation | 226 | CD44 MEFV BID IGF1 MIR146A GAS5 RAC1 ILRUN GDF15 DAZAP1 DELEC1 MIR451A CD4 TUG1 CD1D UBA1 RC3H1 GJA1 NMNAT1 SLC39A14 CGAS NFATC1 SNORD15A NPPB F2RL1 SEMA3A BTRC MIR223 TNFSF10 HAS2-AS1 MIR193A C3AR1 IL24 TLX1NB MBTPS1 TNFSF15 TERC CELF6 MIRLET7C CCL7 SERPINA1 IL1RL1 LTBR CXCL8 EGR1 CCN6 IL27RA SMAD5-AS1 CCL27 IL17A BMP6 TBX21 PPARGC1A TNFAIP6 NCOA3 DEFB4A CBLB ZC3H12A IL23A C5 IL1A LEF1-AS1 TNFAIP8L3 LPCAT2 CSF3 CCL20 MIR6873 SPP1 IL22 LINC02605 MAD1L1 BMP7 IRAK3 AGER IL21R ELF4 TLR5 CDC42 IL18 CYP2E1 SEMA7A IL15 TIE1 TAC1 LRRC19 CAMP ICOSLG SELENOS IL3 TGFB1 ACKR2 MYC SOCS3 MST1 MIAT FBN1 S100A8 RPLP2 ZBP1 IL13 MIR21 TNFAIP3 IL12A IGAN1 MIR125A IL27 TNFRSF25 PPT2-EGFL8 PSMD14 PWAR1 ANGPT2 PPP1CA CPA6 ITGAM FASLG SNORD95 GSEC NMUR1 GGT1 SIGIRR IL1RAPL2 LINC01554 INS ABHD5 MYD88 MIR145 RBCK1 MT-RNR2 MIR30B IL2RA C9orf72 CRH IL10 PGF CUL5 NUBP1 RIPK3 SPI1 ACKR1 DANCR IL3RA FADD IL17F SELPLG MIR142 FGF2 XIST CXCL12 TET2 NR5A2 RIPK1 C5AR2 CXCL9 MIR191 ABCB4 TNIP2 ARPC1B PLA2G7 GRN SOCS1 MIR155 SOX2-OT MIR25 CAPN5 IL1RN EIF2AK4 MAFB LOC654780 CXCL10 TSLP CCL5 SFTPA2 LINC-ROR CARMN IL33 NLRP1 FLG MEG3 MIR146B HLA-B ATXN1 ELAVL1 SHARPIN CFB MIR199A1 OTULIN NMU SNHG1 MALAT1 OIP5-AS1 CLEC4A ICOS IL1B LPCAT3 WIPF1 IL12B IL9 MIR381 RNF114 NFKBIA BECN1 LGALS8 HAMP IL21 TREM1 YARS1 APOA1 CX3CR1 LTA PVT1 CRLF2 CASP4 CEBPB METTL3 CCL3 ATG16L1 TNFSF14 NOTCH1 IL1R1 CD40LG HOTTIP SNHG29 THORLNC HMGB1 S100A9 IL11RA |

**Supplementary table S7** The overlap between the top 23 compounds in combination of *GM-CC-AE* as 5:10:15 and target proteins of inflammation and oxidative stress

| Targets | Total | PAB | MGS | GCC | ABZ | LFR | AGC | R31 | 6PC | DAP | MDS | LDP | IBA | 17BW | DML | DGA | MA | DMC | 8HCA | MGT | ACT |
| --- | --- | --- | --- | --- | --- | --- | --- | --- | --- | --- | --- | --- | --- | --- | --- | --- | --- | --- | --- | --- | --- |
| XDH | 6 |  | ✓ | ✓ |  | ✓ | ✓ |  | ✓ |  |  |  |  |  |  |  |  |  |  | ✓ |  |
| ALOX5 | 10 |  | ✓ | ✓ | ✓ | ✓ |  |  | ✓ |  |  | ✓ |  |  |  | ✓ | ✓ | ✓ |  |  | ✓ |
| ELANE | 2 |  |  |  | ✓ |  |  |  |  |  |  |  |  |  |  |  |  |  | ✓ |  |  |
| SELE | 2 |  |  |  | ✓ |  |  |  |  |  | ✓ |  |  |  |  |  |  |  |  |  |  |
| NFE2L2 | 19 | ✓ | ✓ | ✓ | ✓ | ✓ | ✓ | ✓ | ✓ | ✓ | ✓ | ✓ | ✓ | ✓ | ✓ | ✓ |  | ✓ | ✓ | ✓ | ✓ |
| TNF | 5 |  | ✓ | ✓ |  |  |  |  |  |  |  | ✓ |  |  |  |  |  |  | ✓ | ✓ |  |
| MPO | 1 |  |  |  |  |  |  |  |  |  |  |  |  |  |  |  |  | ✓ |  |  |  |
| CASP1 | 7 | ✓ | ✓ | ✓ |  |  |  |  | ✓ |  |  | ✓ |  | ✓ |  |  |  |  | ✓ |  |  |
| TRPA1 | 6 |  |  |  | ✓ |  |  |  |  | ✓ |  |  |  |  |  |  | ✓ | ✓ |  | ✓ | ✓ |
| PTGS2 | 9 |  | ✓ | ✓ | ✓ |  | ✓ | ✓ |  |  |  |  |  | ✓ |  |  | ✓ |  | ✓ | ✓ |  |
| OLR1 | 2 |  |  |  |  |  |  |  |  |  |  |  |  |  |  |  | ✓ | ✓ |  |  |  |
| IL6 | 1 |  |  |  |  |  |  |  |  | ✓ |  |  |  |  |  |  |  |  |  |  |  |
| PARP1 | 5 |  |  |  | ✓ |  |  |  |  |  | ✓ | ✓ |  | ✓ |  | ✓ |  |  |  |  |  |
| MAPK1 | 11 |  | ✓ |  |  | ✓ |  | ✓ |  | ✓ | ✓ |  |  |  | ✓ | ✓ | ✓ | ✓ | ✓ |  | ✓ |
| FOS | 1 |  |  |  |  |  |  |  |  |  |  |  |  |  |  |  |  | ✓ |  |  |  |
| MAPK8 | 9 | ✓ |  |  | ✓ | ✓ |  |  |  | ✓ | ✓ | ✓ |  |  |  | ✓ |  |  | ✓ |  | ✓ |
| ICAM1 | 2 |  |  |  | ✓ |  |  |  |  |  | ✓ |  |  |  |  |  |  |  |  |  |  |
| MAPK3 | 3 |  |  |  |  |  |  |  |  | ✓ | ✓ |  |  |  |  |  |  |  |  | ✓ |  |
| TP53 | 3 | ✓ |  |  |  |  |  |  |  |  |  |  |  | ✓ |  |  | ✓ |  |  |  |  |
| SIRT1 | 6 | ✓ | ✓ | ✓ |  |  |  | ✓ | ✓ |  |  |  |  |  |  |  |  |  |  | ✓ |  |
| PTGS1 | 13 | ✓ | ✓ | ✓ | ✓ | ✓ | ✓ |  |  |  |  |  | ✓ | ✓ |  | ✓ |  | ✓ | ✓ | ✓ | ✓ |
| TLR2 | 2 |  |  |  |  |  |  |  | ✓ |  |  |  |  |  |  |  | ✓ |  |  |  |  |
| JUN | 2 |  |  |  |  |  |  |  |  |  |  |  |  | ✓ |  |  |  | ✓ |  |  |  |
| Total | | 6 | 9 | 8 | 10 | 6 | 4 | 4 | 6 | 6 | 7 | 6 | 2 | 7 | 2 | 6 | 7 | 9 | 8 | 8 | 6 |
